# Comparative Genome Analysis of Old World and New World TYLCV Reveals a Biasness toward Highly Variable Amino Acids in Coat Protein

**DOI:** 10.3390/plants12101995

**Published:** 2023-05-16

**Authors:** Deepti Nigam, Ezhumalai Muthukrishnan, Luis Fernando Flores-López, Manisha Nigam, Mwathi Jane Wamaitha

**Affiliations:** 1 Institute for Genomics of Crop Abiotic Stress Tolerance, Department of Plant and Soil Science, Texas Tech University (TTU), Lubbock, TX 79409, USA; 2 Plant Pathology and Plant-Microbe Biology Section, School of Integrative Plant Science, Cornell University, Ithaca, NY 14850, USA; 3 Texas Tech University Health Science Center (TTUHSC), Lubbock, TX 79430, USA; 4 Departamento de Biotecnología y Bioquímica, Centro de Investigacióny de Estudios Avanzados de IPN (CINVESTAV) Unidad Irapuato, Irapuato 368224, Mexico; 5 Department of Biochemistry, Hemvati Nandan Bahuguna Garhwal University, Srinagar 246174, Uttarakhand, India; 6 Kenya Agricultural and Livestock Research Organization (KALRO), Nairobi P.O. Box 14733-00800, Kenya

**Keywords:** Begomovirus, polymorphism, co-evolution, diversity, adaption, host range

## Abstract

Begomoviruses, belonging to the family *Geminiviridae* and the genus Begomovirus, are DNA viruses that are transmitted by whitefly *Bemisia tabaci* (Gennadius) in a circulative persistent manner. They can easily adapt to new hosts and environments due to their wide host range and global distribution. However, the factors responsible for their adaptability and coevolutionary forces are yet to be explored. Among BGVs, TYLCV exhibits the broadest range of hosts. In this study, we have identified variable and coevolving amino acid sites in the proteins of *Tomato yellow leaf curl virus* (TYLCV) isolates from Old World (African, Indian, Japanese, and Oceania) and New World (Central and Southern America). We focused on mutations in the coat protein (CP), as it is highly variable and interacts with both vectors and host plants. Our observations indicate that some mutations were accumulating in Old World TYLCV isolates due to positive selection, with the S149N mutation being of particular interest. This mutation is associated with TYLCV isolates that have spread in Europe and Asia and is dominant in 78% of TYLCV isolates. On the other hand, the S149T mutation is restricted to isolates from Saudi Arabia. We further explored the implications of these amino acid changes through structural modeling. The results presented in this study suggest that certain hypervariable regions in the genome of TYLCV are conserved and may be important for adapting to different host environments. These regions could contribute to the mutational robustness of the virus, allowing it to persist in different host populations.

## 1. Introduction

The greatest proportion of emerging diseases that pose a threat to agriculture on a global scale are transmitted by insects, with those transmitted by whiteflies (Hemiptera: *Aleyrodidae*) being the most prevalent. Among the whitefly-transmitted viruses, BGVs (*Geminiviridae*), criniviruses (*Closteroviridae*), and torradoviruses (*Secoviridae*) are considered the most destructive. Begomoviruses (BGVs) are DNA viruses that are categorized as monopartite or bipartite, having circular single-stranded DNA genomes (also known as DNA-A and DNA-B), and being encapsulated in twinned icosahedral capsids [1,2]. The prevalence of monopartite BGVs is greater in the Old World (OW), while the number of reported cases in the New World (NW) is relatively low [2,3]. Being one of the largest genuses, it currently consists of 424 species that infect monocots or dicots, including domesticated and wild plants with devastating loss to agricultural production in tropical and subtropical regions. BGVs rely on the obligate transmission by an insect vector, primarily whitefly species, such as *Bemisia tabaci*, or other whiteflies [3,4], which facilitates their quick and effective spread due to the insect’s indiscriminate feeding behavior. The *B. tabaci* whitefly complex comprises over 35 cryptic species that cannot be distinguished morphologically or by traditional classification methods, and this complex is capable of transmitting over 200 species of BGVs [5,6]. This leads to many potential interactions in nature, with over 200 species of BGVs, more than 35 cryptic *B. tabaci* species, and hundreds of crop species and varieties [7]. Therefore, BGVs are fast-evolving DNA viruses due to the widespread distribution of their whitefly vector and the movement of plant materials across the globe, which is often driven by human activities [8]. The genome size is roughly 2600-nt for each of them [8]. Geographically distinct environment and diverse host plants, in combination with diverse population of insect vectors, can create a heterogeneous selection pressure in virus populations [9]. Despite the limitations in selection, BGVs can infect a diverse range of host plants and insect vectors, and new species are constantly emerging. This indicates that the BGV genome is highly adaptable to mutations and could adjust to new host plants and insect vectors from various regions across the globe [10].

Similar to RNA, DNA viruses also undergo genetic rearrangements during mixed infection that allow interchange of corresponding genes or gene segments [11]. This recombination and reassortment incidents allow the most competent and globally adapted permutations of genes to emerge from the existing genetic pool, thus increasing the potential for viral survival. Previous studies have revealed a prominent intra- and interspecific diversity of BGVs, which can accelerate adaptation to new or changeable climates and novel hosts [12]. On the other hand, certain studies have suggested that BGVs may evolve at a rate comparable to that of RNA viruses solely through mutation [13,14]. Furthermore, positive selection on mutations or the byproducts of recombination events may also contribute to the evolutionary dynamics of BGVs [15,16]. In addition, the utilization of various hosts may also play a significant role in the genetic diversity present in BGV populations [17,18].

The biological environment for each infected individual is subject to fluctuations over time, and viruses that infect a broad range of hosts or are transmitted by multiple vector species may experience diverse environments and distinct selection pressures. Moreover, plant viruses that replicate in both plants and their insect vectors are subjected to markedly different selection pressures in each host, and host selection is a factor in every stage of the virus life cycle [19]. Selection pressure plays a crucial role in maintaining intrinsic properties of virus proteins, such as functional structures that are essential for virus replication [20].

The evolutionary divergence of BGV is complex and involves various factors, such as recombination, mutation, and gene flow [2]. Recombination plays a crucial role in the evolution of BGVs, as it allows for the exchange of genetic material between different strains of the virus [17]. This process can lead to the emergence of new strains that may have different biological properties. The evolution of monopartite and bipartite BGVs may differ due to their distinct genomic organization. For example, bipartite BGVs may have a higher rate of recombination between their DNA-A and DNA-B components, which can result in the emergence of new hybrid viruses. However, monopartite BGVs may have a greater capacity for genetic variation within their single genome, which can lead to the development of new strains through mutation. In summary, the evolution of monopartite and bipartite BGVs is a complex process that involves multiple factors. While both types of viruses can undergo recombination and mutation, their distinct genomic organization may result in different patterns of evolution. Previously, geographically interrelated antigenic variation in 13 BGVs was evidence of vector selection [21]. In addition, selection of some variants from a population of Cucumoviruses extracted by aphid may also be indication of vector selection [22].

Selection of the viral variants depends on their successful transitivity and stability at protein level. The occurrence of geminiviral diseases is frequently linked to two factors: the high genetic variation observed within viral populations and the occurrence of mixed infections where dispensable viral protein interact and evolve at the same time [23,24]. Thus, coevolution plays an imperative role in the stability and interaction pattern of the protein [25]. Consequently, it is also crucial to know the coevolutionary pattern of the viral protein [26,27]. Coevolution assists to fix a favorable mutation of one amino acid with compensatory mutation of another amino acid or groups of amino acids [28,29,30]. Besides, it indicates which amino acids work under evolutionary pressure to make a protein fit for its environment [29,31].

Categorization of genetic variation is crucial to our understanding of virus evolution and host adaptation. Previous studies have shown the differences in the nucleotide diversity in CP and replication protein (Rep) between the OW and NW begomoviral species [32]. This raises the question that if there was entry of BGVs from the OW into the NW and there is high similarity at genomic level within genus, then what evolutionary factors are responsible to drive the higher infectivity in case of BGVs of the OW?

The distinction between OW and NW BGVs is based on their geographic distribution and reflects the evolutionary history of these viruses. It is believed that these two groups arose because of geographic isolation, with OW BGVs being mainly found in the regions of Africa, Asia, and Europe, while NW BGVs are predominantly found in the Americas. The separation of these groups likely occurred a long time ago, and it is thought that their divergence was driven by various factors, including geographical barriers and differences in the host plant populations. While OW and NW BGVs share some genetic similarities, they have evolved independently over time, leading to differences in their genomes. Despite these differences, OW and NW BGVs are still considered to be the same species due to their ability to recombine and exchange genetic material. Furthermore, studies have shown that some OW and NW BGVs can infect the same host species, indicating that they may have similar biological interaction properties.

Several approaches have been used to control BGVs and other viruses in crops, including the use of resistant cultivars, the introduction of resistance (R) genes, RNA interference (RNAi), recessive genomic mutational methods, and pesticides to control the insects’ vectors [33,34]. The CRISPR-Cas (clustered, regularly interspaced short palindromic repeats, CRISPR, associated protein), a bacterial adaptive immune approach against interfering foreign nucleic acids has developed as an effective genome editing technology that has been successfully applied in many organisms, including several plant species [35,36,37]. Nevertheless, intra and inter-genomic variation and virus evolution signal include the escapee characterization from CRISPR-Cas9 plants engineered to target BGV genomes [38]. However, targeting two or more sites simultaneously in the viral genome with multiple sgRNAs inhibit geminiviral accumulation and generate viral escape [39,40,41].

Comparing the OW and NW BGVs can provide insights into the mechanisms driving virus evolution and adaptation to different environments. For example, comparing the genetic diversity and recombination patterns between these two groups can reveal how different environmental factors have shaped their evolutionary trajectories. Additionally, studying the host ranges and interactions of these viruses with their insect vectors can provide insights into the co-evolutionary processes driving virus–host interactions.

To evaluate the model, we classified *Tomato yellow leaf curl virus* (TYLCV) into categories based on their occurrence in OW and NW geographical regions by parsing the GenBank files, as the virus is found in both regions. SNPs were identified from both groups, and their distribution was analyzed throughout the genome of TYLCV. The findings demonstrated that hypervariable regions are present in different parts of the genome in TYLCV from both OW and NW regions. Comparative analysis between TYLCV isolates from the OW and NW revealed that the highest variation was observed in the replication-associated protein (Rep), C3, C4, and coat protein (CP), with sites under positive selection. Notably, the degree of variation was significantly greater in TYLCV isolates from the OW.

Given Begomoviral CP vital importance, both in terms of their infectivity and antibody-based resistance [42,43,44,45,46], we felt a serious need for the analysis to evaluate its evolution. Thus, our primary objective was to identify and describe the patterns of mutations that indicate positive selection in the coat protein (CP) of both OW and NW viruses, as well as to assess how these patterns change over different geographical regions. The present study’s results indicate the presence of promising mutation sites under positive selection (fast-evolving sites) within the loop region of CP from OW TYLCV isolates. Interacting amino acids within the coat protein of OW TYLCV isolates have been identified for the first time, followed by subsequent evolutionary analysis. These mutations appear to be accumulating across different regions of OW and could be a significant determinant of the virus’s ability to adapt to a wide range of host plants and insect vectors. Further research may be necessary to understand the specific functional implications of these mutations and their role in the evolution and adaptation of OW TYLCV.

Additionally, in this study, we conducted a variation analysis and identified several sites that are positively selected. One of these sites, S149N (78%), was found to have originated in China but rapidly spread to Europe and underwent structural changes. Moreover, we detected a densely clustered group of amino acids in the CP of OW viruses, which may represent a new evolutionary paradigm. The increased structural flexibility of the CP may provide mutational robustness and allow for the maintenance of biological functions. Consequently, mutations in the viral coat protein sequences, particularly at specific sites, could lead to alterations in vector-dependent transmission, which could increase the likelihood of the emergence of a resistant viral strain [47,48]. The identification of these sites in the CP of TYLCVs could serve as promising functional targets for CRISPR/Cas9 gene editing and need further investigation.

## 2. Results

### 2.1. Characteristics of BGVs Genomic Sequences

A total of 12,332 and 3943 genomic DNA sequences for OW and NW bipartite genomes, respectively, were obtained from NCBI virus database https://www.ncbi.nlm.nih.gov/labs/virus/vssi/#/ (accessed on 30 August 2021). To allow for comparison, only the DNA-A component was considered. Out of the total 269 OW species and 153 NW species identified as full-length DNA-A sequences, only these were included for further analyses. The average length of DNA-A sequences from both groups ranged from 1950 to 2067 nucleotides in length. Chargaff’s purine–pyrimidine equilibrium was detected in both groups with a ratio of 1.0, and the average purine content (G + A) was 49.82% for OW and 49.47% for NW, while the pyrimidine content (U + C) was 50.17% and 50.53%, respectively, with no significant difference. The GC percent was identified as 43.17% for OW and 43.89% for NW.

### 2.2. The Nucleotide Diversity Observed in BGVs from the Old World Differs from That Seen among New World BGVs

**Figure 1:**
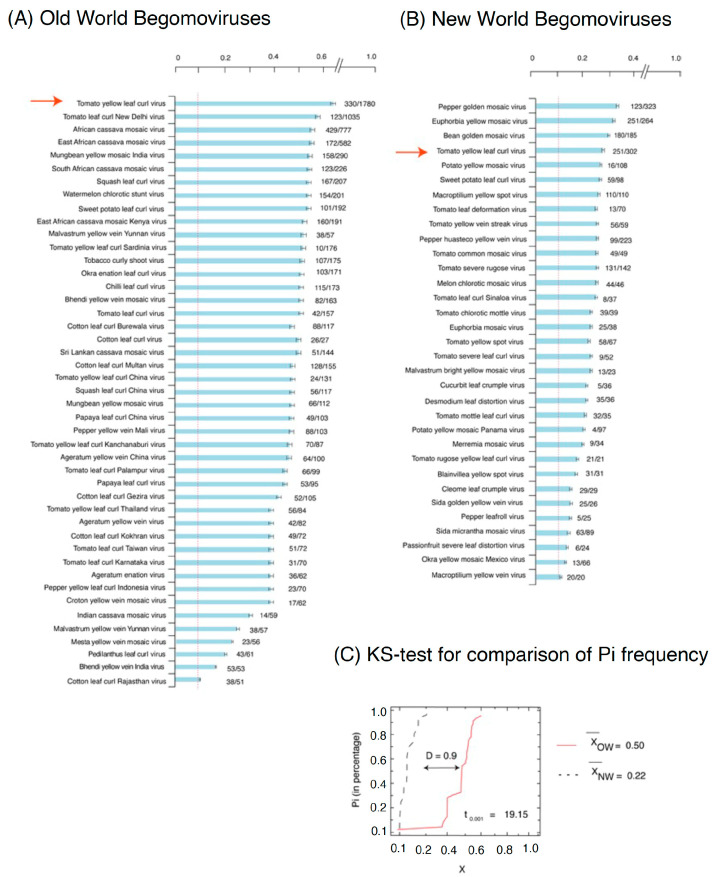
Nucleotide diversity (Pi) in begomovirus genomes of species from the Old World (OW) in (**A**) and New World (NW) in (**B**). Full-length genomes for 269 monopartite OW species and 153 NW species were obtained from the NCBI database and evaluated (DNA-A component only). The scale bar at the top shows the nucleotide diversity (in percentage) calculated for each virus species indicated on the left side of each histogram. Only species with nucleotide diversity values of 0.1% or higher are represented in the graph. The bars of the histogram represent the fraction of normalized polymorphic sites (number of single nucleotide polymorphisms per length of the genome) for each virus. To the right of each bar is indicated the number of full-length nucleotide accessions analyzed/total number of sequence accessions present in the database. Bars represent the average and standard error for each species, analyzed for 50-nt intervals over the entire genome. The vertical, red-dotted line represents the 99% confidence interval for measurable nucleotide variation. Red arrows point to *Tomato yellow leaf curl virus*, the only species present in both populations with significant genomic variation. (**C**) Kolmogorov-Smirnov test (KS-test) comparison plot showing deviation (D = 0.9 at *p*-value = 10^−23^) in Pi frequency distribution from OW and NW species. Cumulative distributions of percentage of nucleotide diversity (in%) in the genome of OW and NW species. The D values printed inside the plot symbolize the degree of deviation of the plot from the OW and NW species curve. The larger the D value, the greater the difference in their genomic variations. The X_OW_ and X_NW_ represent the calculated mean value of Pi for BGVs from the OW and NW, respectively. The t denotes a Student’s t-value (Student *t*-test statistic) for significance at *p*-value 0.001. Degree of freedom (d.o.f) was calculated as 55 at critical value 2.01. The genomic variation in begomoviral species from the OW is much higher than those from the NW (*p*-value ≤ 0.001).

The nucleotide diversity (pi) method [49] was found to be preferable over other methods for analyzing genetic diversity in virus species due to the unequal distribution of accessions or isolates in each virus species. This method measures nucleotide substitutions and corrects for the number of accessions, providing a more accurate estimate of genetic diversity in virus populations [50]. Results showed that 44 of the 269 OW species exhibit a higher Pi (Figure 1A). Across these 44 OW species, TYLCV was top ranking, with the highest genomic variation, and 60% of sites were variable i.e., Pi = 0.6. Other OW species with significant variation in their genome were *Tomato leaf curl New Delhi Virus* (TLCNDV), African cassava mosaic virus (ACMV), *East African cassava mosaic virus* (ACMV), and *Mung bean yellow mosaic India virus*, with Pi values of 0.58, 0.55, 0.55, and 0.54, respectively, which means that >50% of the nucleotide positions in their genome are polymorphic. However, 33 NW counterpart species showed significant genomic variation in their genome with highest variation (Pi = 0.29) identified in *Pepper golden mosaic virus* (PGMV) (Figure 1B). The other NW species identified were *Euphorbia mosaic yellow mosaic virus* (EYMV), *Bean golden mosaic virus* (BGMV), TYLCV, and *Potato yellow mosaic virus*, with Pi’s of 0.29, 0.29, 0.26, and 0.25. Overall, the Pi analyses for the DNA-A revealed that mean nucleotide diversity of BGVs from the OW is more than twice that observed for BGVs from the NW (Figure 1A,B). A significant deviation (D = 0.54, *p*-value = 0) is shown by the Kolmogorov-Smirnov test in (Figure 1C). Comparing the begomoviral species between OW and NW suggest that 14 species are commonly present in both regions, such as TYLCV, *Ageratum yellow vein virus* (AYVV), *Clerodendrum golden mosaic China virus* (CGMCV), *Corchorus yellow vein virus* (CYVV), *Cotton leaf curl Gezira virus* (CLCGV), *Sida yellow mosaic virus* (SYMV), *Squash leaf curl virus* (SLCV), *Sweet potato golden vein associated virus* (SPGVV), *Sweet potato leaf curl Georgia virus* (SPLCGV), *Sweet potato leaf curl Sao Paulo virus* (SPLCSPV), *Sweet potato leaf curl Spain virus* (SPLCSV), *Sweet potato leaf curl virus* (SPLCV), *Sweet potato mosaic virus* (SPMV), and *Watermelon chlorotic stunt virus* (WCSV). In parallel to comparison of similar components, i.e., DNA-A across OW and NW species, DNA-B was compared with the DNA-A from both groups. Results obtained from our analysis suggest that DNA-B component is more variable than the DNA-A component of NW species (Supplementary Figures S1 and S2). As TYLCV has been identified in both OW and NW geographical regions and known as having the widest host range, we pursued a more detailed comparison of the genomes of TYLCV isolates from the OW and NW.

### 2.3. The Distribution of Nucleotide Diversity in Tomato Yellow Leaf Curl Virus Varies across the Genome and Differs among Old World versus New World Isolates

**Figure 2:**
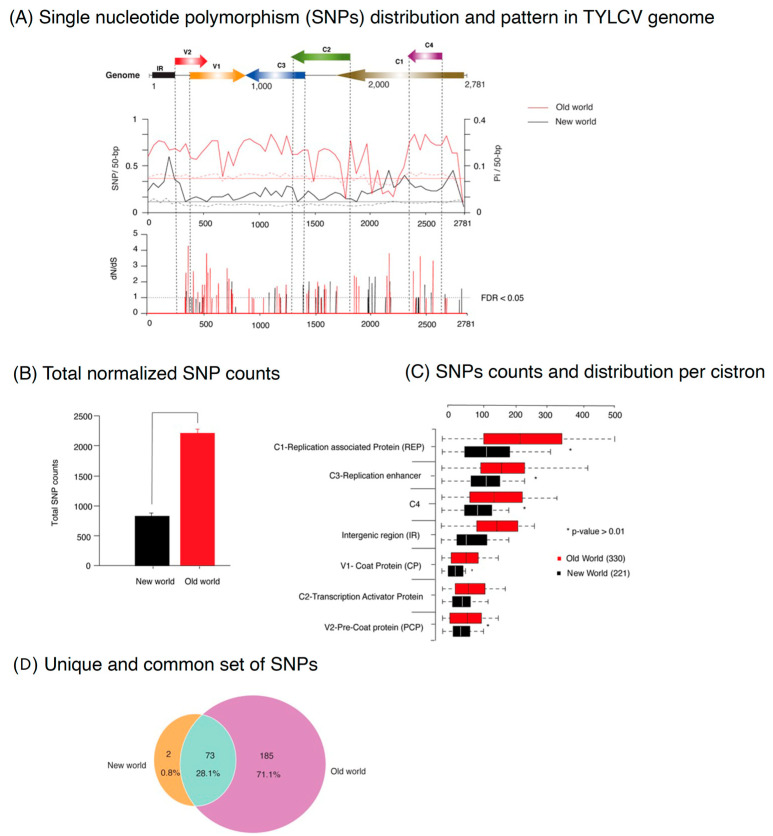
Genomic variation identified in *Tomato yellow leaf curl virus* (TYLCV) isolates from Old World (OW) and New World (NW) geographical regions and their comparison. (**A**) SNP and nucleotide diversity (Pi) distribution and pattern in TYLCV genome. The genomic organization of TYLCV is shown at the top, with the open reading frame and intergenic region (IR) identified. SNPs were estimated and normalized in a 50-nt window (SNPs/50-bp) in an analysis of full-length TYLCV genomes of 330 OW accessions (red line) and 221 NW accessions (black line). The X-axis corresponds to genomic coordinates (nucleotides) of the consensus genome derived after the alignment and Y-axis denotes SNP counts. The plot at the bottom shows the dN/dS, the ratio of the number of nonsynonymous substitutions per synonymous substitution encoded at positions along the TYLCV genome shown above. Red and black lines denote positive selection sites in the OW and NW isolates, respectively. The dotted line represents the threshold value above which sites are under positive selection with a false discovery rate (FDR) of 5%. (**B**) Comparison of the number of normalized SNP counts per cistron (normalized based on cistron length) among TYLCV isolates derived from the OW versus the NW, red and black bars, respectively. (**C**) Histogram showing the normalized total TYLCV genome SNP counts separately for of isolates from the OW and NW; a chi-square test shows a significant difference (*p*-value < 0.01) in the SNPs. (**D**) A Venn diagram showing the number and proportion of unique and common SNPs in TYLCV genomes from the OW and NW. Purple color shows unique SNPs in genomes from the OW. Orange represents a unique number of SNPs in genomes from the NW (i.e., 2 SNPs; 0.8%), while the set of SNPs in common between the NW and OW genomes is shown in light green.

Genetic variability in viral proteins is often influenced by the surrounding environment and the range of hosts available [51]. TYLCV has been reported to have the broadest host range among the BGVs, infecting at least 49 plant species from 16 different families [52], and it is distributed across both the OW and NW geographic regions. To discover and characterize the distribution of mutations in the TYLCV genome separately from OW and NW, single nucleotide variation (SNPs) and nucleotide diversity (Pi) were estimated on a 50-nt window and mapped to each genomic segment of DNA-A (Figure 2A). Moreover, for the cistrons in each segment, positive selection analyses were executed using two independent methods, i.e., SLAC and MEME [53]. The codon sites predicted by both methods were considered under selection, as already reported by previous studies [54,55,56]. Mutations in the virus genome could be dispensed randomly or restricted to form hypervariable areas. In TYLCV, nucleotide variation is not uniformly distributed between genomic DNA-A segments and not randomly distributed within each segment (Figure 2A). OW isolates of TYLCV have a higher number of SNPs in their genomes than NW isolates with a mean of 0.35 SNPs/50-bp for OW isolates versus 0.17 for the NW isolates (chi-square *p*-value ≤ 0.001) (Figure 2B). This is also seen in the values for the normalized total TYLCV genome SNP counts (Figure 1C). The pattern of SNPs in OW isolates show at least four regions of conservation, with depressed numbers of SNPs relative to the average (in V1, C2, C1, IR). By contrast, the pattern of SNPs in NW isolates shows on average, a relatively low level of SNPs/50-bp, with three regions of higher variability (in the IR, C1/C4, and C1). As described above, for both OW and NW isolates, the frequency of SNPs is not the same in all cistrons; the counts are higher in the C1 cistron than in other cistrons (chi-square *p*-value ≤ 0.001).

Selection analysis based on dN/dS (or the ratio of nonsynonymous to synonymous substitution rates, >1, with *p*-value ≤ 0.05) displayed the presence of higher number of codons sites under positive selection and especially prominent in the V1, C1, and C1/C4 cistrons (Figure 2A). A lower number of sites under positive selection were detected in C3 in both OW and NW (Figure 2B). Irrespective of origin, comparison across the genome from both groups revealed a common pattern, where Rep genes harbor the highest variation, followed by C3, C4, and CP genes (Figure 2C). However, a contrasting variation pattern across the isolates from both groups might indicate different sources of selection constraints imposed by vectors, hosts, and possible roles of different parts of the genome in host and vector adaptation.

The subsequent variation analysis yielded a total of 258 SNPs in OW TYLCV isolates and 75 SNPs in NW isolates (Figure 1B, right panel). To characterize the difference at qualitative level of SNPs between TSWV isolates of OW and NW origin, we performed a Venn analysis. Most of the SNPs (total 185; ~71%) were unique to OW isolates, while only two (0.8%) were unique to the NW counterpart (Figure 2D). In total, 28.1% were common in both groups. A separate analysis was performed to measure the transition and transversion ratio between the genome of OW and NW TYLCV isolates. Results revealed a significant amount of C to T and G to A transitions (Supplementary Figure S3).

### 2.4. Amino Acid Substitution Profile in Coat Protein of TYLCV

**Figure 3:**
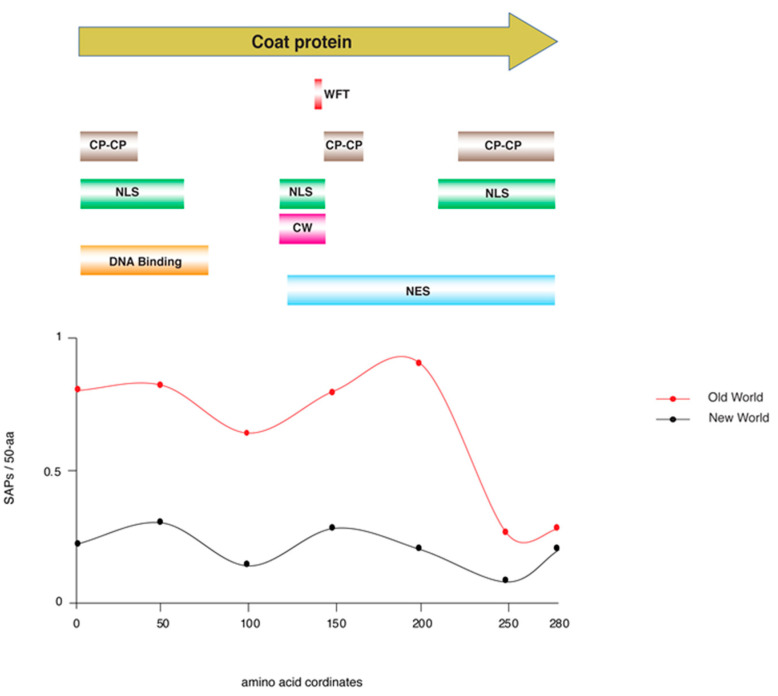
Counts of single amino acid polymorphisms (SAPs) in the coat protein (CP) of TYLCV. Counts of SAPs per 50 amnio acid window (SAP/50-aa) are shown on the y-axis; those of TYLCV isolates from the Old World (OW) are indicated in red and those from New World (NW) isolates are in black. CP amino acid coordinates from 1 to 258 are shown along the x-axis. At the top, the CP is shown diagrammatically as a tan arrow from the N- to the C-terminus, left to right. Functional domains are highlighted below the corresponding regions of the CP. These include white fly transmission, WFT; CP–CP binding, CPCP; nuclear localization signal motifs, NLS; a cell wall targeting motif, CW; DNA binding, and a nuclear export signal domain, NES.

Understanding genetic polymorphism in viruses is a fundamental need in order to elaborate on virus epidemiology and evolution [57,58,59]. Our previous analysis showed that there is a difference in SNP accumulation per cistron in the genome of OW and NW TYLCV isolates (Figure 1B,C). Begomovirus coat protein (CP) is a multifunctional protein known to interact with vectors for successful transmission [60]. A high degree of variation in the different viral determinants are known to provide a potential to adapt to and overcome changing environments [51,61]. Although genetic variation for determinants of RNA virus has been well documented to impact virus–host interactions, how it alters the interactions in DNA virus, insect vector, and plants is still mysterious. Here, by the aid of in-silico analysis, we identified single amino acid polymorphism (SAP) in the coat protein of TYLCV isolates from OW and NW (Figure 3). The findings indicated that SAPs exhibit an uneven distribution, with a substantial level of diversity in CP observed in the OW, whereas the NW showed comparatively low levels of variation. To see which regions of CP are under high variation, we further analyzed the pattern of SNP distribution. Results suggested a high polymorphism (a peak) within the region of 150–200 amino acid (aa) window that encompasses three functional domains, nuclear localization signal (NLS), central nuclear export signal (NES) domain (orange color), and the cell wall targeting motif (CW) (Figure 3). The in vivo studies have shown that the molecular interaction between CP of TYLCV within the window of 81–222 amino acid residues and whitefly egg vitellogenin enables transovarial virus transmission [62,63]. After normalizing with the length of CP from both groups, in total, 225 SAPs were identified in the genome of the OW whereas only 71 were present in the NW (*p*-value < 0.01) (Figure 3). This significant difference in the degree of amino acid variation in CP implies that a differential evolutionary pressure may act on the TYLCV genome from both groups and might enable them to adapt competently to the varying vector population.

**Figure 4:**
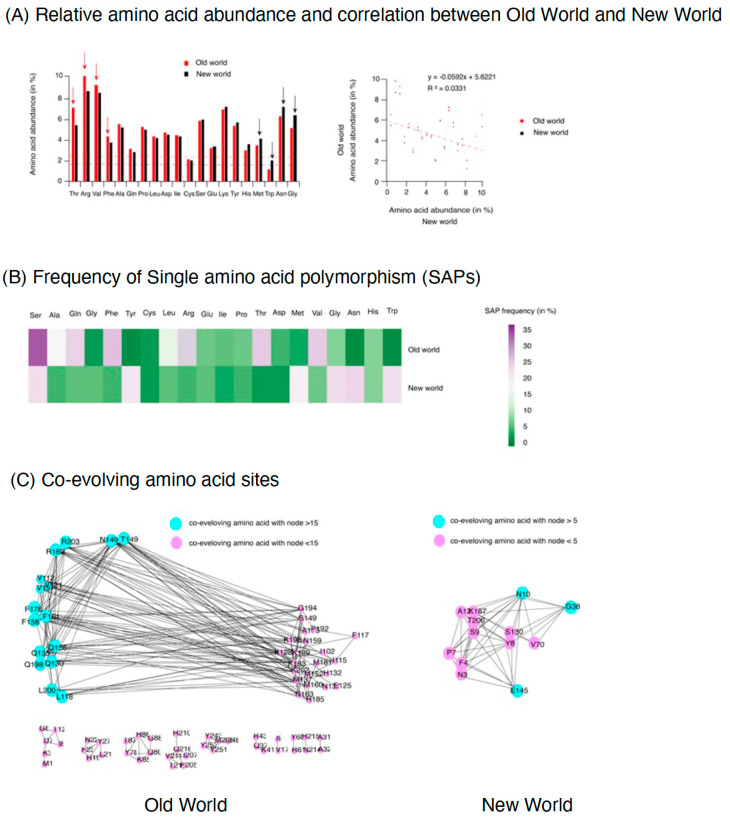
Dynamics of amino acids in the coat protein (CP) of Old World (OW) and New World (NW) TYLCV. (**A**) Relative amino acid abundance and comparison for CP in TYLCV isolates from OW and NW geographical regions. Bar chart (left panel), showing relative abundance of 20 amino acids in coat protein of OW(red bars) and NW (black bars). X-axis is the amino acids and Y-axis is the relative abundance (calculated in percentage). Red and black dotted lines are the means calculated for both groups, respectively. Higher abundance (significant at *p*-value 0.05) is shown with arrow relative to each other (red arrow OW and black arrow NW). Left panel showed a Pearson correlation plot for comparison in relative amino acid abundance with calculated R2 value = 0.03. (**B**) Heat map showing the frequency of single amino acid polymorphism (SAPs) calculated in in coat protein of OW (left panel) and NW (right panel) TYLCV isolates. The amino acids are shown on top with frequency rate represented as colored scale of 0 (green) to 35 (purple). (**C**) Highly coevolving amino acid identified (with bootstrap value of 100) in CP of OW (left panel) and NW(right panel) TYLCV isolates. The Blue nodes show high co-evolving amino acids with higher connecting partner with neighbor nodes > 15 in OW and >5 in NW. The pink color nodes represent coevolving amino acids with neighborhood nodes < 15 in OW and <5 in NW.

We examined the amino acid content at each protein and profiled amino acid substitutions to examine the effect of genomic variation on protein variation. The relative amino acid abundance was calculated for TYLCV isolates from OW and NW. Interestingly, the amino acid abundance differed in each protein (Figure 4A). The most abundant amino acids were threonine (Thr, 6.1%), arginine (Arg, 10%), and valine (Val, 9%) in OW TYLCV isolates with comparatively less abundance in NW. Whereas, glycine (Gly, 7.2%), asparagine (Asp, 6.5%), and tryptophan (Trp, 2%) were higher in NW isolates, but lower in OW. However, there was a very poor correlation in the relative abundance between the isolates from both regions (R2 = 0.003). With the amino acid substitution analysis, frequent substitutions identified were observed for Thr (35%), Arg (32%), Val (25%), and Phe (23%) in the isolates of OW. While, in NW, the frequently mutable amino acids were Gly (34%), Asp (25%), and Trp (26%) (Figure 4B). Previous research has indicated that the amino acids glycine and arginine can contribute to the structural disorder of viral proteins [64]. In contrast, tryptophan promotes protein stability [65] and was the least abundant amino acid in OW. Thus, in TYLCV, the pattern of amino acid abundance and substitution follows the same pattern for the proteins of the same group (OW and NW), but they differ from each other. Further, to test the evolutionary pressure in CP from the isolates of both geographical regions, co-evolution was identified (Figure 4C). Result showed that CP in OW isolates contain groups of highly co-evolving amino acids, such as Thr at position 149, Asp at position 149, Arg at 203 and 182, Val at 112 and 152, and Phe at 178 and 158 in the CP of OW TYLCV. Whereas, only three, Asp at position 10, Gly at 38, and Glu at 145, were identified in NW isolates (Figure 4C). The results imply that certain amino acids at these positions are under strong selection pressure and exhibit bias.

### 2.5. Amino Acid Mutation in Coat Protein from Old World TYLCV Isolates Are Structure Changing

**Figure 5:**
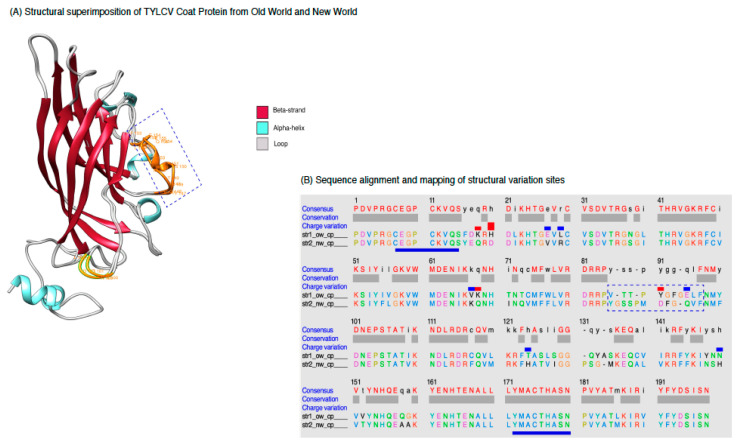
Structural modeling of the TYLCV coat protein (CP) based on the cryo-EM structure of ageratum yellow vein virus (AYVV) (PDB ID: 6f2s.1.J) CP. (**A**) Superimposition of CP model of an Old World (OW) isolate (ALN12562.1, blue color) and a New World (NW) isolate (QCG7473.1, brown color) of TYLCV. Structural deviation between two CP structures was detected and heighted in orange color. The blue dotted regions denote a major structural change, i.e., from loop (in OW) to alpha helix (in NW). A small helix region (MDFG) is observed in the NW isolate, but a loop region (PYGF) is observed in the OW region. (**B**) Alignment of the amino acid sequences of the two modeled CPs (from panel (**A**)), sharing 80% identity. The amino acids that are underlined as dark blue show the motifs, which are recognized by a whitifying vector (*Bemisia tabaci*). The sequence with the dotted box as dark blue color are the amino acids that are part of the loop region (149–159 bp) in OW TYLCV isolate, while changed, while the alpha helix is the NW isolate (as in panel (**A**)).

To evaluate the effect of mutation in CP structure of TYLCV from both groups, the mutation was analyzed using Phyre2 (Protein Homology/analogy Recognition Engine V 2.0) server (http://www.sbg.bio.ic.ac.uk/phyre2/html/page.cgi?id=index) (assessed on 20 August 2021) [66]. The CP models were created and visualized using Chimera, version 2 (Figure 5). Structure-based alignment of TYLCV CP sequences suggest that there are differences in the amino acids located in the window from 149 to 159. These amino acids are surrounded by a conserved peptide that was previously known to affect vector transmission [67,68] (Supplementary Figure S4). During our observations across the BGVs, we detected a significant number of SAPs, including a substantial amount of deletions (Supplementary Figure S5). Specifically, the analysis of the CP protein structure for the OW isolate ALN12562.1 and the NW isolate QCG7473.1 demonstrated that most of the differences in amino acid composition are concentrated within the loop region (Figure 5A,B). These variations in the loop region are believed to be under strong positive selection and may reflect the evolutionary pressure imposed by the diverse host plants and insect vectors that these viruses encounter. Additionally, the mutations identified in these flexible loop structures are consistent with the constraints on virus evolution and adaptation to different environments. Further, CP structure superimposition suggests an emergence of new short, small helix region (MDFG) in the NW isolate, but a loop region (PYGF) in the OW. It might be due to differences in functional requirements, such as interaction with different host factors (new vector adaptation or new host) [17,69]. Selected models for CP in OW and NW demonstrated accurate topology, as governed by their C score, expected TM-score, RMSD value, as well as stabilization of its stereo-chemical properties [70]. Stability of CP structures was further confirmed by Ramachandran plot statistics that showed a low percentage of amino acid residues to have phi/psi angles in disallowed regions [71]. Further superimposition of refined protein models of OW and NW resulted in a RMSD value of 0.561 Angstroms (Å) and revealed major variations in the secondary structure of the protein, i.e., loop in OW to alpha-helix regions in NW that resulted in local protein conformational changes from its native to the mutated form (Table 1). These structural variations might be associated with interaction of TYLCV CP with diverse host factors.

### 2.6. The S149 Mutation: Increasing Frequency and Worldwide Distribution

**Figure 6:**
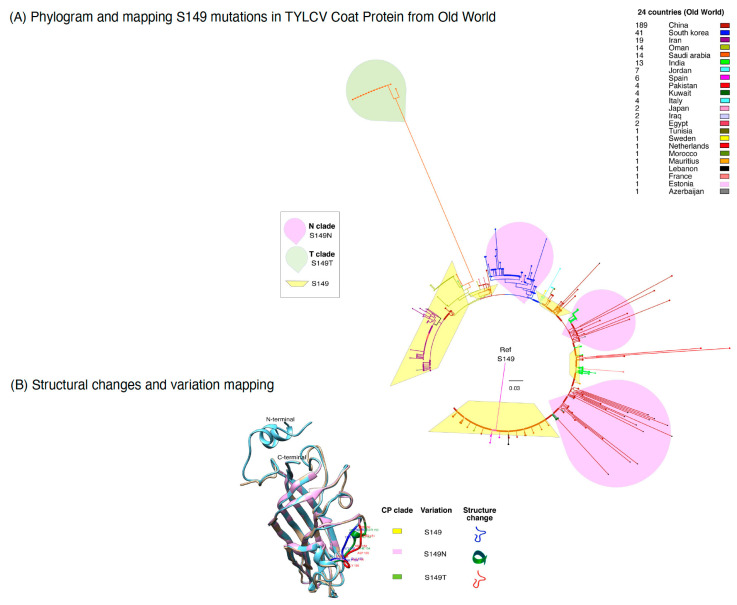
Phylogenetic trees based on 330 coat protein (CP) full length alignments from TYLCV Old World (OW) isolates retrieved from NCBI. (**A**) A basic neighbor joining tree, centered on the TYLCV reference strain from Spain. Clades (N and T) were divided based on the mutations of serine at position 149. N clades (named for the S149N mutation) are highlighted in pink. T clades (named for the S149T mutation) are highlighted in orange. The regions of the world where sequences were sampled are indicated by colors. An interesting pattern of coat protein mutations that we are tracking are against the backdrop of the phylogenetic tree based on the full genome. Note three distinct patterns: mutations that predominantly emerge to be part of a single lineage S149N are found in very different regions, both geographically and in the phylogeny, indicating the same mutation seems to be independently arising and a mutation S149T in sequences from the same geographic location, but arising in very distinct lineages in the phylogeny (orange) found only in Saudi Arabia. The tree shown here was made using MAFFT software. (**B**) The amino acid variation mapped in the loop region of coat protein (CP) model from OW and NW TYLCV isolates. The corresponding geographical regions are color-decoded, as in panel (**A**).

While we embarked on CP analysis, our inspiration was to distinguish mutations that might be of potential concern. In a situation of very low genetic diversity, conventional methods of identification of functional mutations have imperfect statistical power. However, a rich NCBI data set offers a possibility to look more deeply into the evolutionary relationships among the TYLCV sequences in the context host and geography (Figure 6). We found that, at position 149 of the CP reference sequence (NC_004005) from Spain, Almeria contains serine (S), while in many other isolate sequences of the OW, such as S at position 149, the results changed to asparagine (N) (78%). Interestingly, in some TYLCV sequences originating from Saudi Arabia, a new mutation was observed, where S is changing to Threonine (T), i.e., S149T. Superimposition of these coat protein structures reveals a deviation in the same region (149 to 159), but, in the new helix structure, the coat protein of TYLCV isolates from the OW. However, the loop structure was maintained in the coat protein of NW TYLCV.

This methodology of backtracking the mutation over phylogeny revealed that TYLCV isolates bearing the coat protein mutation S149N or S149T are replacing the original Spain form of the virus rapidly and repeatedly across the globe (Figure 6A). We do not know what is compelling this selective sweep. The S149N change causes a new helix formation and thus is consistent with several hypotheses regarding a fitness advantage that might be explored experimentally. S149N is embedded in the region which has upstream and downstream peptide motif effecting vector transmissibility. Accordingly, this mutation might be conferring change in interaction with new host factor (vector and plants) from different geographical regions. Finally, the S149N mutation is predicted to have direct consequences for the infectivity of the virus and might be consistent with rapid spread in different regions of Europe and Asia (78%). S149T is potentially interesting because of a very different evolutionary trajectory (Figure 6A). It is only found in a single lineage in Saudi Arabia and could be a new strain in future.

**Figure 7:**
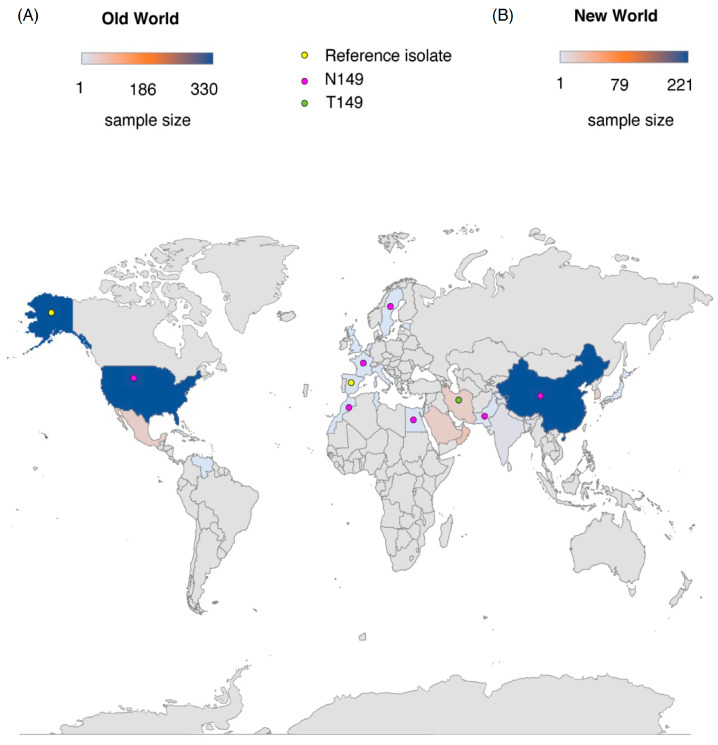
Global mapping and distribution of the single amino acid polymorphisms (SAPs) at position 149 in coat protein of TYLCV isolates from (**A**) the New World (NW; 221 isolates) and (**B**) the Old World (OW; 330 isolates). The sample size for analysis from different regions are represented by the color scale from light blue to dark blue (scale shown above). The SAPs are indicated with colored dots on the map.

To know if there is a structural diversity in CP of OW isolates, we divided the sequence into three clades with respect to reference strain from Spain, i.e., S-clade, N-clade, and T-clade (Figure 6B). Accordingly, a structural superimposition analysis was performed for three CP sequences that were representative of these clades. In concordance with the phylogeny in Figure 6A, isolates that have similarity to reference sequence types, i.e., serine at position 149 (S-clade) or the S149T mutation (T-clade), contain loops within windows 149 to 159. Whereas a short helix structure was found for the isolates that have the S149N mutation (N-clade) in CP (Figure 6B). The identified S149 amino acid polymorphism in OW and NW TYLCV isolates were also mapped to worldwide locations (Figure 7B).

### 2.7. The S149 Mutation and TYLCV Evolution Are Linked to Host Geography

**Figure 8:**
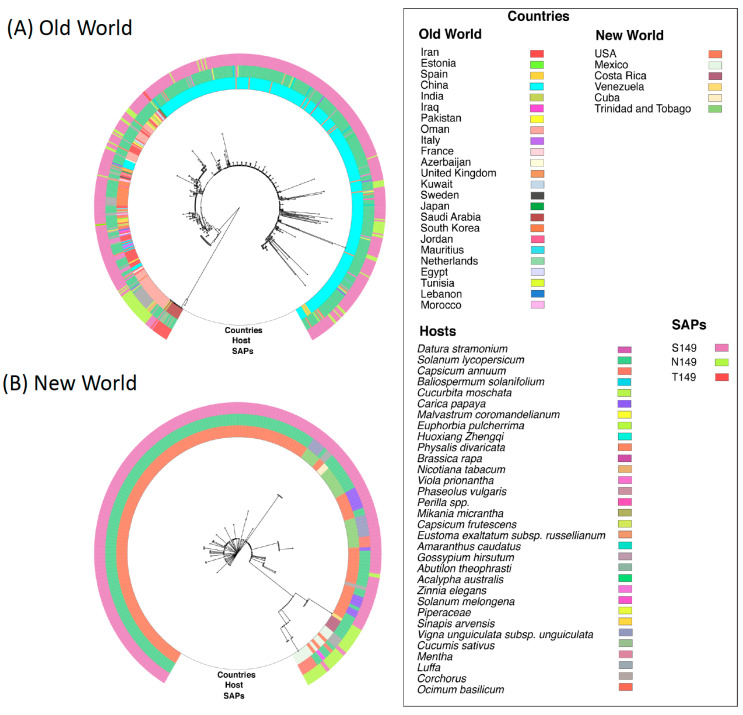
Phylogeography showing evolving clades of *Tomato yellow leaf curl virus* (TYLCV) isolates based on coat protein amino acid sequences. Isolates were identified as coming from geographic regions in either (**A**) the Old World (OW) or (**B**) the New World (NW) and grouped on this basis into two data sets for analyses. The phylogenetic trees were generated using full-length coat protein sequences and the Python-based program GraPhlAn. The position of virus isolates was aligned with colored outer rings to indicate the country of origin, the plant host, and the single amino acid polymorphisms (SAP) at position 149. Serine (S149) was the most common amino acid at this position, whereas T149 was the least common and not observed in NW isolates.

The inherent variability observed in the coat protein (CP) of TYLCV implies its significant involvement in the process of evolution and diversification. To investigate this further, we constructed separate phylogenetic trees using the CP sequences of OW and NW TYLCV, which allowed us to compare the evolutionary patterns of the two groups. By analyzing the CP protein sequences of both groups, we gained a better understanding of the evolutionary history of TYLCV and how it has evolved over time. To create the phylogenetic trees, we added information about the host plant species and the country of origin of the TYLCV isolates. The resulting phylogenies had different structures, with the OW phylogeny showing more clades and branches compared to the NW phylogeny. This suggests that the CP of OW TYLCV has accumulated more mutations over time than the CP of NW TYLCV. Additionally, the OW isolates showed higher nucleotide diversity and a greater range of host types, suggesting that maintaining flexibility in host adaptation is an important factor in TYLCV evolution. Interestingly, the host geography appeared to be a more important factor in determining the clustering of TYLCV isolates than the host plant species. In contrast, the NW isolates showed less genetic divergence, indicating a more recent common ancestor compared to the OW isolates. This could be due to a smaller population size, lower mutation rate, or more recent emergence of the virus in the region. These findings support the idea that mutations in the CP are responsible for the emergence of new TYLCV strains and are a major contributor to its evolution. However, further research is needed to fully understand the evolutionary patterns of TYLCV in different regions. (See Figure 8A,B for the corresponding phylogenetic trees).

## 3. Discussion

Plant viruses often rely on biological vectors to spread from one plant to another in nature. Among them, the Begomovirus genus, which belongs to the Geminiviridae family, is particularly remarkable due to its large size in the virosphere and its ability to infect a broad range of plant species. Moreover, they can be transmitted non-persistently by over 420 species of aphids, which highlights their remarkable ability to adapt to different environments, vectors, and hosts. In addition, the impact of climate change is expected to heighten the incidence of viral epidemics as vectors expand their range into previously unaffected regions, which could lead to the exposure of new hosts to the virus. As every interaction between host, vector, and environment has the potential to eliminate unfit viruses, it is crucial for viruses to maintain their functionality and a high level of fitness to survive within the population. The current study examined the genome-wide variation between isolates of OW and NW TYLCV (one of the highly infecting BGVs) and identified hypervariable regions of the genome that exhibit a preference for nucleotide substitutions and positive selection (as shown in Figure 2, Figure 3, Figure 4, Figure 5, Figure 6, Figure 7 and Figure 8). Furthermore, our study has provided insight into the role of coevolving amino acids in the CP proteins, which may contribute to host adaptation by increasing the flexibility of the protein. We have discussed the specific mutations found in each cistron of TYLCV, providing a comprehensive analysis of their potential functional significance.

### 3.1. Mutation Dynamics and Selection Constraint in Begomovirus Cistron; TYLCV as Model

There are several odd practices in the virosphere when it comes to genome partitions in plant viruses. However, the exact reasons for the presence of multipartite genomes in plant viruses are still not fully understood, and this remains an area of active research [72,73]. Among ssDNA genomes, and in the virosphere as a total, Begomovirus is the utmost widespread multipartite genus, characterizing a core impact on the global abundance of multipartite species. The degree of evolutionary similarity across different genomic components of segmented and multipartite viruses varies on the interaction between evolutionary and biological processes operating at both the inter- and intragenomic levels [70]. Hence, diverse structural and functional constraints along genomic regions might drive such genomic regions and even whole segments to distinct patterns of evolution. Though, usually, different segments should follow a similar evolutionary way, assuming the functional dependence among segments, previous studies have shown that different components in multipartite viruses might experience distinct evolutionary routes [74,75].

Results obtained with our in-silico analysis demonstrated a different evolutionary pattern, with significant differences in variation that DNA-A segment of OW and NW BGVs, DNA-A segment, and DNA-B segments of NW BGVs, as well as across different cistrons level (Supplementary Figure S3). To investigate the dynamics of nucleotide substitutions in the BGVs, we undertook a genome wide variation analysis in one of the most devastating and well studied tomato pathogens, *Tomato yellow leaf curl virus* (TYLCV) [76,77], for which a comparatively ample data set of whole-genome sequences subsist. A detailed outcome of variation analysis for each cistron are discussed below.

### 3.2. Intergenic Region (IRs)

The two-virion sense open reading frames (ORFs); CP/V1 and V2 and four complementary sense ORFs, i.e., C1, C2, C3, and C4, are interspaced by an intergenic region (IRs), which comprises a sequence capable of forming a stable hairpin loop structure, including the motif 5′-TAATATTAC-3′ common to all geminiviruses. The intergenic region is furthermore recognized as a site of the origin of DNA replication (ori). This region is particularly prone to variation and specifies a sensitive guide to differences between isolates. Previous analysis has shown that IRs in *Tomato leaf curl viruses* [78] and other similar geminiviruses are highly divergent and evolve speedily with a mean substitution rate of ~1.56 × 10^−3^ subs/site/year [79]. Our analysis also supports a high number of nucleotide variation (SNPs) in TYLCV genome from OW and NW (Figure 1). In OW isolates, the variation peaks at N-terminal region, while in the NW counterpart, it is prominent at the C-terminal.

### 3.3. V2

The V2 from BGVs encodes a multifunctional protein, which is required for full infection and to suppress gene silencing at the transcriptional (TGS) and post-transcriptional (PTGS) level [80,81]. Furthermore, it has been described that the V2 protein is necessary for virus movement and transmission throughout the plant [82]. Additionally, it is identified to be involved in the regulation of host defense responses and it provokes symptoms of hypersensitive response (HR)-like cell death phenotype in *Nicotiana benthamiana* plants [83]. The V2 protein (known as AV2 for bipartite BGVs) is conserved across the BGVs from the OW, but it is absent from viruses originating from the NW [84]. In previous reports, mutation of the PKC motif of AV2 of the bipartite BGV, EACMCV, diminished pathogenicity and its ability to suppress RNA silencing within hosts [85,86]. For PaLCuV, as well as V2, deletion in this motif abolishes the ability to induce hypersensitive response (HR) [87]. Our variation analysis across OW and NW TYLCV isolates suggests a contrasting pattern in the V2 gene, where, within OW, the SNP peaks are at the middle region, while, in NW, it is higher—at the N-terminal region.

### 3.4. Coat Protein/V1

BGVs are mainly transmitted by their whitefly vector, and the CP is the only protein that is exposed to whitefly tissues with specific motif, enabling interaction with insect receptors or other proteins that assist or enable virus translocation into or through insect tissues [88]. The CP serves multiple functions in the viral life cycle, particularly in the context of whitefly-mediated transmission. CP helps in regulating the levels of viral accumulation within the whitefly vector [89]. This balance is crucial for efficient transmission. Too much viral replication may overwhelm the whitefly’s defenses, while too little replication may hinder successful transmission to new plants. Despite its structural role in the viral capsid, phylogenetic studies have shown that the CP gene sequences are quite divergent due to a high mutation rate [90]. Previous studies have shown that the CP region is highly susceptible and tolerant to drastic amino acid changes, affecting viral pathogenesis [91]. Therefore, CP mutations could potentially expedite novel means of transmission, such as seed transmission [92]. In our present study, we observed a dissimilar pattern of SNP distribution in TYLCV CP between OW and NW isolates, with a higher number of SNPs in OW compared to NW isolates. Additionally, we investigated the varying abundance of certain amino acids in OW isolates, along with their co-evolving counterparts.

### 3.5. C3 (Replication Enhancer Protein)

The C3 protein is known to enhance the replication of geminivirus DNA accumulation. Despite its known ability to enhance geminivirus DNA replication, studies have indicated that even with mutant C3 replicons, replication can still occur, although to a lesser degree, in both single cells and plants [93]. Interestingly, most of the mutations identified did not affect the replication enhancement activity of C3 in tobacco protoplasts [94]. Some mutations even boosted or impaired C3 replication activity. Our comprehensive analysis of mutations in the C3 protein of TYLCV could provide new insights into improving understanding behind its replication efficiency.

### 3.6. C2

The AC2 protein of BGVs is highly conserved and plays a critical role in viral infectivity [95]. Inactivating mutations in the AC2 gene have been shown to abolish the expression of important viral genes, rendering the virus non-infectious [96]. Therefore, the AC2 protein can be considered a virulence factor, as it is an infectious component that damages the host. In our analysis, mutations identified in the C2 cistron of TYLCV may provide insight into the high virulence and wide host range of the virus.

### 3.7. C1

The replication-associated protein (Rep) encoded by the AC1 ORF (also called AL1) in bipartite geminiviruses and by C1 (also called L1) in monopartite geminiviruses (except mastreviruses) is known to be conserved in sequence, position, and function and is expressed under the control of a bidirectional core promoter in the IR [82]. The Rep protein is crucial for rolling circle replication (RCR) and is involved in the modulation of gene expression [97], comparable to some animal and bacterial ssDNA viruses and plasmids, signifying a robust evolutionary connection between these proteins. The profiling of single nucleotide polymorphisms (SNPs) in the C1 region of OW *Tomato yellow leaf curl virus* isolates has the potential to impact its replication in diverse host environments. This, in turn, can play a significant role in the virus’s adaptation and evolution.

### 3.8. C4

C4, also identified as symptom determinants, has drawn much attention in recent years. Being the tiniest and one of the least-conserved proteins encoded by geminiviruses [98], C4 proteins may establish the highest number of functions in infection cycles and pathogenesis, with novel functions continuing to be identified [99]. C4 proteins may provoke the abnormal development of plants by controlling the brassinosteroid (BR) signaling pathway through interactions with members of SHAGGY-like protein kinase [100]. C4 proteins of some specific BGVs have been shown to be a viral suppressor of RNA silencing [101]. Additionally, some key amino acids in BGVs C4 proteins have been shown to be involved in the modulation of the severity of leaf curling symptoms [99]. Our analysis showed that a high number of SNPs in the C4 region of OW TYLCV isolates, which may indicate its potential role in providing flexible functional sites that can interact with host factors, potentially leads to changes in symptom severity or other aspects of the virus’s infection cycle. Further investigation may be necessary to better understand the specific functional implications of these SNPs and their involvement in the adaptation and evolution of OW TYLCV.

In summary, the results of this study suggest that the Rep, C1, C3, and C4 proteins of OW TYLCV isolates may play critical roles in the virus’s adaptation and evolution in different host environments. The novel discovery of co-evolving amino acids in the coat protein region of TYLCV isolates from OW underscores the significance of investigating the dissemination of OW TYLCV isolates. These differences may enhance their adaptability to host plants and vectors, contributing to their extensive host and vector range. The identified variation profiles in this study could also have important practical applications, including the development of diagnostic tools for BGVs, breeding BGV-resistant crops, and identifying genes susceptible to BGVs. Further research is necessary to fully understand the functional implications of these variations and their potential impact on virus-host interactions.

## 4. Material and Methods

All the computation work was performed on high-performance computing nodes of BioHPC (https://biohpc.cornell.edu/lab/lab.aspx) (assessed on 20 August 2021) at Cornell University, Ithaca, NY, USA. Scripts created specifically for this study are available upon request.

### 4.1. Genomic and Polyprotein Sequences

The complete genome or full-length open reading frame (ORFs) of BGVs species represented in GenBank (http://www.ncbi.nlm.nih.gov/) (assessed on 20 August 2021), using custom-made scripts available under Entrez Programming Utilities (E-utilities; https://www.ncbi.nlm.nih.gov/books/NBK25500/ (assessed on 20 August 2021). The GenBank files were parsed based on the geographical location, and species were categorized as OW and NW groups. For each species, an accession defining the complete genome, and coordinates for each cistron, were used as a reference genome (Supplementary Table S1 and Figure S1). Accessions having less than 95% of the reference genome length were omitted. For significant statistical assessments, only the species with at least three accessions were included [51]. *In-house* bioperl and perl scripts were developed to evaluate the purine, pyridine, and GC percentage from the consensus sequence for each begomoviral species.

### 4.2. Single Nucleotide Polymorphism (SNP) Analysis

The genomic sequence alignment (.aln) file were separately obtained for OW and NW isolates with multiple sequence alignment (MSA) using MAFFT (software https://mafft.cbrc.jp/alignment/software/) (assessed on 20 August 2021). These alignment files in nexus format for all genomic sequences were used to establish pairwise nucleotide diversity (Pi) in a 50-nt sliding window using the Tajima’s D test in DnaSP 5.10.1 [102]. In an alternating approach, the same alignment files were used for identification of variation pattern, i.e., SNPs, using SNP-sites version 2.4.1 (https://github.com/sanger-pathogens/snp-sites) (assessed on 20 August 2021). The type i.e., Transition (Ts) or Transversion (Tv), and position of each nucleotide substitution, were mined in a variant call format (VCF). By exploiting a method, --SNPdenisty, from the package VCFtools [103], SNPs were obtained in a 50-nt or amino acid window and normalized to the length of the window. Consequently, for each virus and group, i.e., OW and NW, a variation index was calculated by normalizing total SNPs to the length of corresponding virus genome. To resolve a dissimilarity threshold in both analyses, a 99% confidence interval was assessed using the Z-score [X ± (Z × s × √n)], as described previously [51].

### 4.3. Kolmogorov-Smirnov Test

To determine the statistical significance difference between two non-Gaussian, cumulative distributions of nucleotide diversity (Pi) between the species of OW and NW, we quantified the difference using the D-statistic of the two-sample Kolmogorov-Smirnov (KS) test [104]. D-statistics were calculated using the R function ks test [105]. All the values of the D-statistic calculated showed significant differences between the two worlds, with *p*-value in the range of 10^−23^.

### 4.4. Discovery of Coevolving Groups in Coat Protein

Full length nucleotide and protein sequences of each begomoviral species corresponding to NCBI GenBank accessions (Supplementary Table S1) were used for the analysis. The multiple sequence alignment (MSA) of these sequences was made using default parameters of MAFFT [106]. The phylogenetic tree of these MSA was inferred with PhyML [107] and used for coevolution detection, as implemented in BIS2Analyzer (http://www.lcqb.upmc.fr/BIS2Analyzer/submit.php) (assessed on 20 August 2021) [108] and CAPS version 2 (http://caps.tcd.ie/) (assessed on 20 August 2021) [109]. Amino acids were weighted based on biochemical properties, namely, Grantham [110], polarity [111], charge [112] and dipeptide bonds [113]. The statistical significance (*p*-value ≤ 0.05) of identified coevolving sites and false discovery rate were evaluated [114]. Each coevolving amino acid residue in multiple sequence alignment (MSA) was interpreted and visualized as an amino acid interaction network using Cytoscape v3.9.0 [115].

### 4.5. Coat Protein 3-D Structure Prediction, Validation, Visualization, and Analysis

A custom bash script was used to extract the CP amino acid sequence based on the coordinate information in GenBank records. The three-dimensional structure of the CP of the two TYLCV isolates viz. ALN12561.1 from OW and QCG74731.1 from NW with the most variation was generated using Phyre2 (http://www.sbg.bio.ic.ac.uk/phyre2/html/page.cgi?id=index) (assessed on 20 August 2021) under intensive mode [116]. Predicted CP models were subjected to Structural Analysis and Verification Server (SAVES) (http://services.mbi.ucla.edu/SAVES) (assessed on 20 August 2021) for evaluation and quality checking. Models (in.pdb format) for two viruses or isolates were superimposed using Chimera v1.13 (https://www.cgl.ucsf.edu/chimera/ (assessed on 20 August 2021) [117]. The TM-Score was used for structure alignment measurement [118].

## Figures and Tables

**Table 1:** Statistics of CP (V1) protein structural predictions from TYLCV of Old World (OW) and New World (NW) (C score: confidence score, TM score: template modeling score, RMSD: root mean square deviation).

Structure Modeling Features		Old World -CP (ALN12561.1)	New World -CP (QCG74731.1)
Top 10 templates predicted by I-TASSER		1i3yA, 1i4yA, 1i6zA, 6cmlA, 6cmnA, 6i9yA, 8cmlA, 8cmnA, 1i5yA, 1i7zA	1i2yA, 1i2yA, 1i6zA, 8cmlA, 7cmnA, 4i9yA, 4cmlA, 4cmnA, 1i4yA, 9i7zA
Model Evaluation data of predicted structures	C- score	−2.86	−2.23
	Expected TM score	0.29 ± 0.10	0.61 ± 0.12
	Expected RMSD	15.5 ± 2.4	14.8 ± 2.9
	Number of Decoys	322	412
	Cluster Identity	0.0143	0.02
Energy value (KJ/mole) of predicted protein models	Before energy minimization	−1048.821	140.521
	After energy minimization	−14,412.217	−12,125.214
Ramachandran Plot statistics (% residues)	Favored regions	89	88.9
	Additional region	8	7
	Allowed regions	2	3
	Disallowed regions	1	1.1

## Data Availability

All datasets for this study are included in the article/Supplementary Material.

## Supplementary Materials

The following supporting information can be downloaded at: https://www.mdpi.com/article/10.3390/plants12101995/s1, Table S1. Nucleotide and polyprotein features of Begomoviruses used in this study. Figure S1. Comparison of Nucleotide diversity (Pi) in between the DNA-A and DNA-B component of begomovirus species from the New World (NW) in (**A**) Full-length sequences of DNA-A and DNA-B component for 26 bipartite NW species were obtained from NCBI database and evaluated. The scale bar at the top shows the nucleotide diversity (in percentage) calculated for each virus species indicated on left side of each histogram. Only species with nucleotide diversity values of 0.1% or higher are represented in the graph. The bars of the histogram represent the fraction of normalized polymorphic sites (number of single nucleotide polymorphisms per length of the genome) for each virus. Red bars denote DNA-A component, while black bar to DNA-B. Bars represent the average and standard error for each species, analyzed for 50-nt intervals over the entire genome. To the right of each bar, is indicated the number of full-length nucleotide accessions analyzed/total number of sequence accessions present in the database. The vertical, black-dotted line represents the 99% confidence interval for measurable nucleotide variation. (**B**) Kolmogorov-Smirnov test (KS–Test) comparison plot showing deviation (D = 0.9 at *p*-value = 10–23) in Pi frequency distribution in between the DNA-A and DNA-B component of NW species. Cumulative distributions of percentage of nucleotide diversity in the DNA-A and DNA-B component. The D values printed inside the plot symbolize the degree of deviation of the plot from the OW and NW species curve. The larger the D value, the greater is the difference in their genomic variations. The XOW and XNW represent the calculated mean value of Pi the DNA-A and DNA-B component, respectively. The t denotes a student t value (student test statistic) for significance at p–value 0.001. Degree of freedom (d.o.f) was calculated as 34 at critical value 2.01. The genomic variation in begomoviral species from the DNA-A is much higher than those from the DNA-B (*p*-value = 0.001). Figure S2. Nucleotide diversity (Pi) in DNA-B component of begomovirus species from the New World (NW). Full-length genomes for 153 NW species were obtained from NCBI database and evaluated. The scale bar at the top shows the nucleotide diversity (in percentage) calculated for each virus species indicated on left side of each histogram. Only species with nucleotide diversity values of 0.1% or higher are represented in the graph. The bars of the histogram represent the fraction of normalized polymorphic sites (number of single nucleotide polymorphisms per length of the genome) for each virus. To the right of each bar, is indicated the number of full-length nucleotide accessions analyzed/total number of sequence accessions present in the database. Bars represent the average and standard error for each species, analyzed for 50-nt intervals over the entire genome. The vertical, red-dotted line represents the 99% confidence interval for measurable nucleotide variation. Figure S3. Nucleotide substitutions determined for full length genome of Tomato yellow leaf curl virus (TYLCV) isolates from (**A**) S (OW) and (**B**) New World (NW) geographical regions. The proportion of substitution was compared within each group and evaluated with chi-square test (X2) tests of significance. The expected number of substitutions are shown as grey bar, while observed number of substitutions are in red. Star shown on the top bars represent significance (*p*-value) of the chi-square value. Note: expected number of nucleotide substitution is calculated by adding the total forward and reverse of every substitution type (i.e., the sum of the number of G to T and T to G transversions), assuming they were equally likely by dividing that sum in half, and then correcting that value by the relative frequency of each base in the deduced TYLCV reference genome sequence (i.e., occurrence of base T/[occurrence of base T + occurrence of base G]) (Nigam et al., 2019 [51]). Figure S4. The coat protein structure model and comparison between Old World and New World. (**A**) The CP structure from AYVV (left, in blue) and TYLCV (right, in green). (**B**) Structure superimposition of CP from AYVV and TYLCV. (**C**) The ribbon diagram at 0o, 90 o and 180 o rotation. (**D**) Illustrations of functional motifs such as WFT (White fly transmission), CP-CP c-terminal and CP-CP- middle domain and nuclear export signal (NES) in CP. Figure S5. Alignment of Coat Proteins for Begomoviral and Single amino acid polymorphism (SAPs).
