# Supplementary material for: Comparative Genome Analysis of Old World and New World TYLCV Reveals a Biasness toward Highly Variable Amino Acids in Coat Protein
Source: Plants (Basel). 2023 May 16;12(10):1995. doi: 10.3390/plants12101995 (PMC10223811; doi:10.3390/plants12101995)
Supplement: Supplementary file 1 [file plants-12-01995-s001.zip › supplementary figures_march_22_DN.pdf]

A) Comparision of DNA-A and DNA-B in New World Begomoviruses

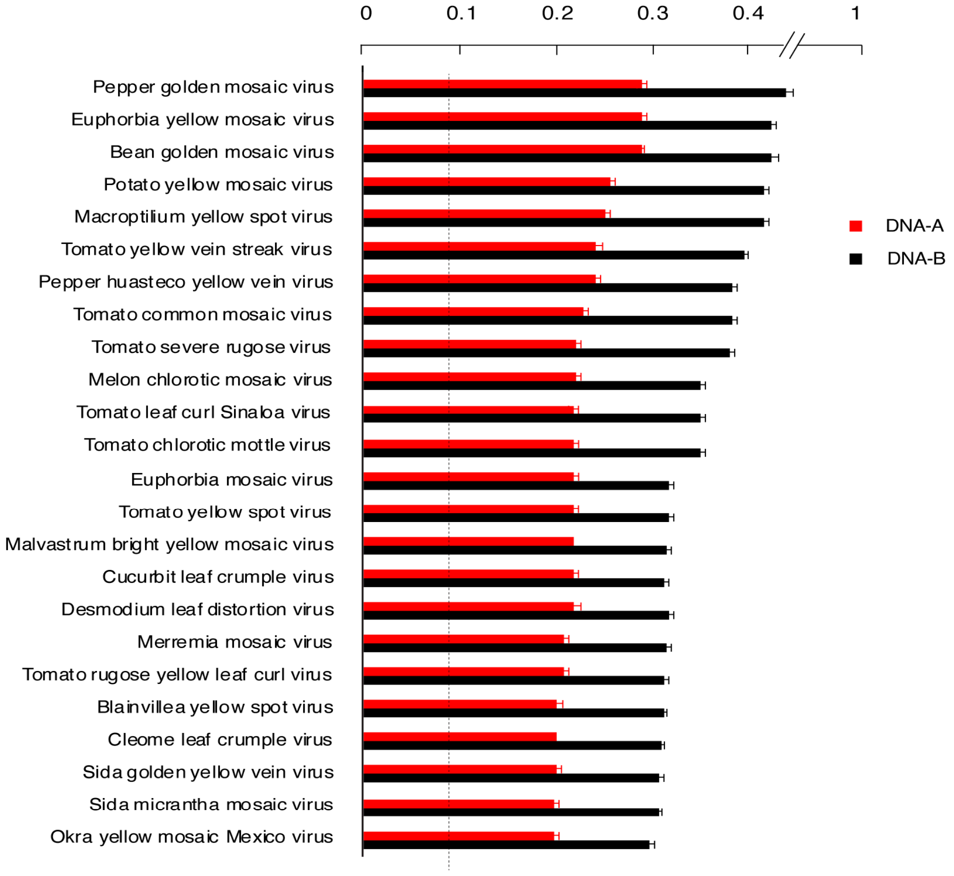

B) KS-test for comparison of Pi frequency

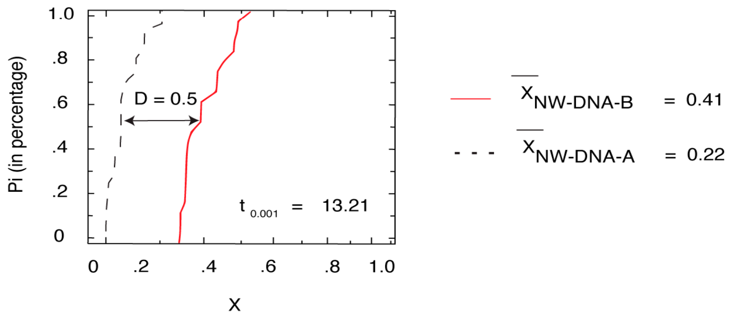

Supplementary Figure S1. Comparison of Nucleotide diversity (Pi) in between the DNA-A and DNA-B component of begomovirus species from the New World (NW) in (A) Full-length sequences of DNA-A and DNA-B component for 26 bipartite NW species were obtained from NCBI database and evaluated. The scale bar at the top shows the nucleotide diversity (in percentage) calculated for each virus species indicated on left side of each histogram. Only species with nucleotide diversity values of 0.1% or higher are represented in the graph. The bars of the histogram represent the fraction of normalized polymorphic sites (number of single nucleotide polymorphisms per length of the genome) for each virus. Red bars denote DNA-A component, while black

bar to DNA-B. Bars represent the average and standard error for each species, analyzed for 50-nt intervals over the entire genome. To the right of each bar, is indicated the number of full-length nucleotide accessions analyzed / total number of sequence accessions present in the database. The vertical, black-dotted line represents the 99% confidence interval for measurable nucleotide variation. B) Kolmogorov-Smirnov test (KS –Test) comparison plot showing deviation (D = 0.9 at p-value =10-23) in Pi frequency distribution in between the DNA-A and DNA-B component of NW species. Cumulative distributions of percentage of nucleotide diversity in the DNA-A and DNA-B component. The D values printed inside the plot symbolize the degree of deviation of the plot from the OW and NW species curve. The larger the D value, the greater is the difference in their genomic variations. The XOW and XNW   represent the calculated mean value of Pi the DNA-A and DNA-B component, respectively. The t denotes a student t value (student test statistic) for significance at p–value 0.001. Degree of freedom (d.o.f) was calculated as 34 at critical value 2.01. The genomic variation in begomoviral species from the DNA-A is much higher than those from the DNA-B (p-value = 0.001).

DNA-B of New World Begomoviruses

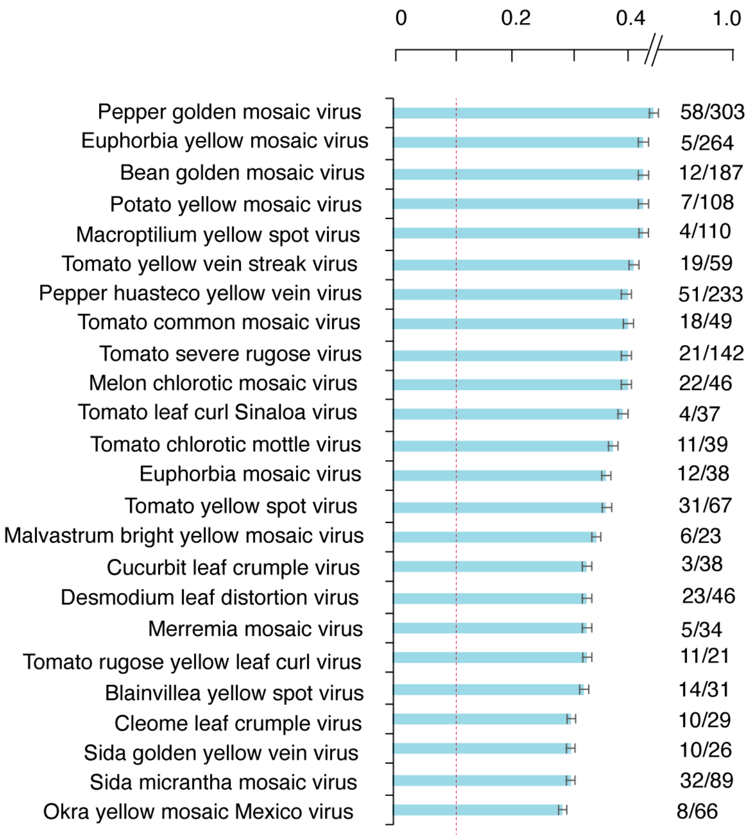

Supplementary Figure S2. Nucleotide diversity (Pi) in DNA-B component of begomovirus species from the New World (NW). Full-length genomes for 153 NW species were obtained from NCBI database and evaluated. The scale bar at the top shows the nucleotide diversity (in percentage) calculated for each virus species indicated on left side of each histogram. Only species with nucleotide diversity values of 0.1% or higher are represented in the graph. The bars of the histogram represent the fraction of normalized polymorphic sites (number of single nucleotide polymorphisms per length of the genome) for each virus. To the right of each bar, is indicated the number of full-length nucleotide accessions analyzed/total number of sequence accessions present in the database. Bars represent the average and standard error for each species, analyzed for 50-nt

intervals over the entire genome. The vertical, red-dotted line represents the 99% confidence interval for measurable nucleotide variation.

A)

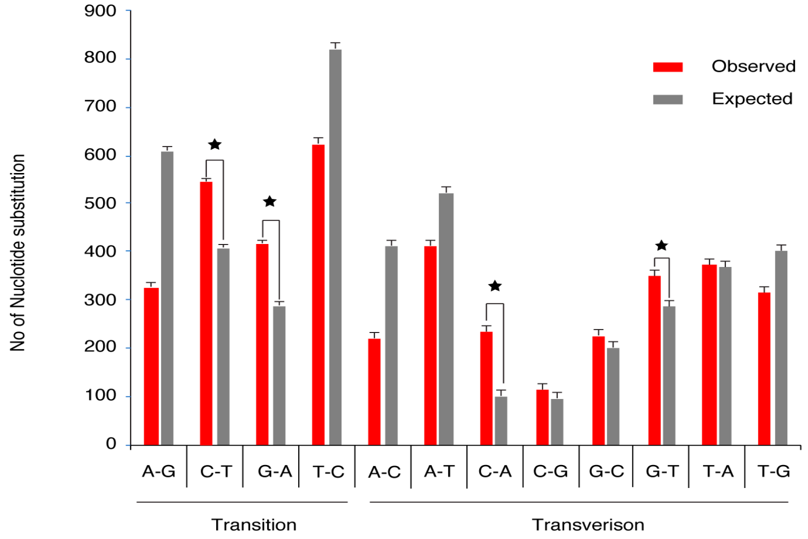

B)

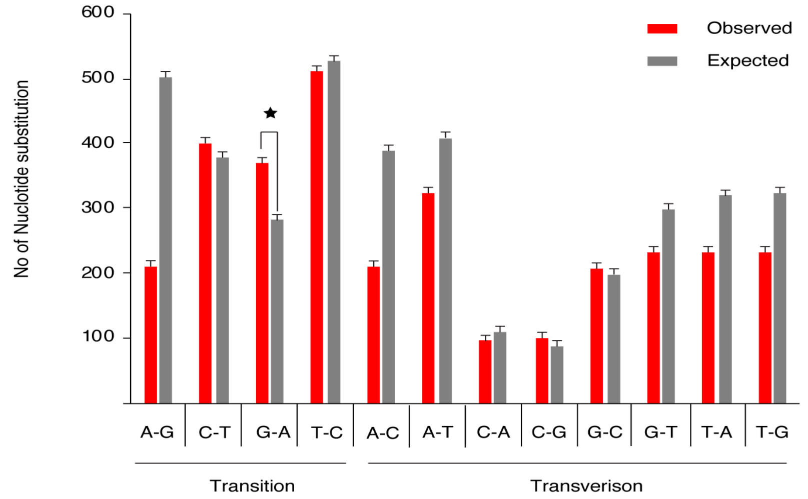

Supplementary Figure S3. Nucleotide substitutions determined for full length genome of Tomato yellow leaf curl virus (TYLCV) isolates from A) Old World (OW) and B) New World (NW) geographical regions. The proportion of substitution was compared within each group and evaluated with chi-square test (X2) tests of significance. The expected number of substitutions are shown as grey bar, while observed number of substitutions are in red. Star shown on the top bars represent significance (P-value) of the chi-square value. Note: expected number of nucleotide substitution is calculated by adding the total forward and reverse of every substitution type (i.e., the sum of the number of G to T and T to G transversions), assuming they were equally likely by dividing that sum in half, and then correcting that value by the relative frequency of each base in the deduced TYLCV reference genome sequence (i.e., occurrence of base T /[occurrence of base T + occurrence of base G]) (Nigam et. al, 2019 [51]).

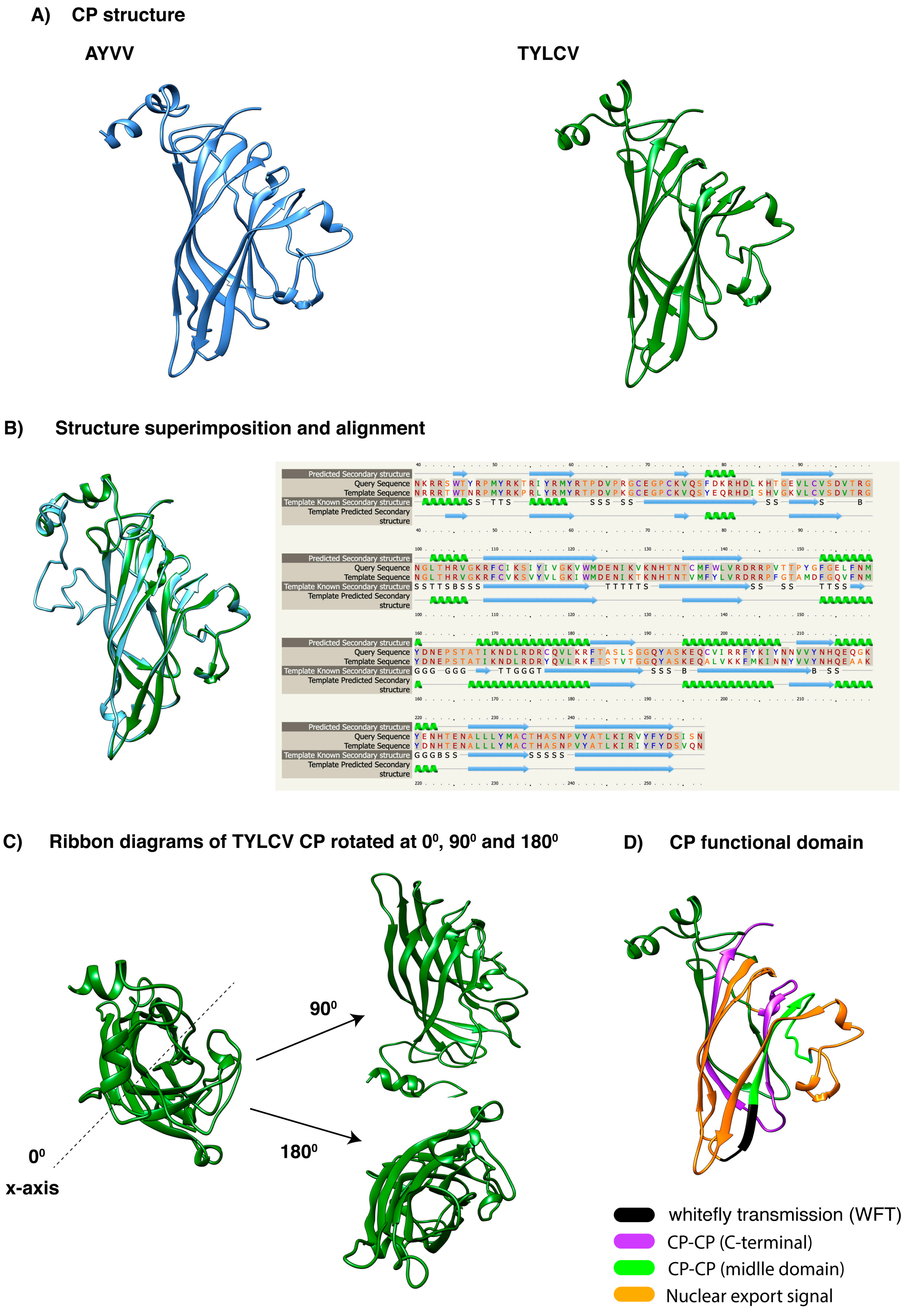
A) CP structure
AYVV
TYLCV
B) Structure superimposition and alignment
Predicted Secondary structure
Query Sequence
Template Sequence
Template Known Secondary structure
Template Predicted Secondary structure
C) Ribbon diagrams of TYLCV CP rotated at 0⁰, 90⁰ and 180⁰
90⁰
180⁰
0⁰
x-axis
D) CP functional domain
whitefly transmission (WFT)
CP-CP (C-terminal)
CP-CP (midlle domain)
Nuclear export signal

Supplementary Figure S4. The coat protein structure model and comparison between Old World and New World. A) The CP structure from AYVV (left, in blue) and TYLCV (right, in green). B) Structure superimposition of CP from AYVV and TYLCV. C) The ribbon diagram at 0°, 90° and 180° rotation. D) Illustrations of functional motifs such as WFT (White fly transmission), CP-CP c-terminal and CP-CP- middle domain and nuclear export signal (NES) in CP.
