# Supplementary material for: Comparative Genome Analysis of Old World and New World TYLCV Reveals a Biasness toward Highly Variable Amino Acids in Coat Protein
Source: Plants (Basel). 2023 May 16;12(10):1995. doi: 10.3390/plants12101995 (PMC10223811; doi:10.3390/plants12101995)
Supplement: Supplementary file 1 [file plants-12-01995-s001.zip › Supplementary Figure_S5.pdf]

```
consensus   1 RRRLNFDS-RRSRARAp-tRRSTNKKRGWSKRPMRRKGRSYRMYRSP--------PLKVQSFEinq--fgPDFVVS---- 64
Q08595      2 RRGAYTPR-STPFPRD---RRSYNAGKGRSFRSYRRRGPVRPLARRNlfg--ddhARAFTYKTlsedqfgPDFTIHn--- 72  Indian cassava mos...
Q17ZR1     18 RRRFNFDSpRPSIAAAl--TAPGTRRRRWTNRPMYRKPRFYRMYRTPdvpkgcegPCKVQSFD-------AKDDIG---- 84  Sida yellow vein v...
Q6ZX39     18 RRRLNFDSpYTSRVAAp-tVLVTNKRKSWVNRPMYRKPRMYRMFRSPdvprgcegPCKVQSFD-------AKNDIG---- 85  Tomato leaf curl C...
Q1JRP0     18 RRRLNFDSpYTGRAVAp-tVRVT-RRQMWSNRPMYRKPMMYRMYRSPdvprgcegPCKVQSYE-------ARHDVS---- 84  Tomato yellow leaf...
Q8JNG6     18 RRRLNFDSpYSSRAAVp-iVQGTNKRRSWTYRPMYRKPRIYRMYRSPdvprgcegPCKVQSFD-------KRHDLK---- 85  Tomato leaf curl S...
Q6L6V9     18 RRRLNFDGpYVSRVNVp-iAQGTSKKRSWAQRPMYRKPRIYRMYRSPdvpkgcdgPCKVQSFE-------KKDDVG---- 85  Honeysuckle yellow...
AWW16572   18 SRNAN----YSPRARIgprV---DKASEWVHRPMYRKPRIYRTLRTAdvprgcegPCKVQSYE-------QRHDIS---- 79  Squash leaf curl v...
O55376     16 RRRLTFDTpRATPASL----GRSNKRRAWENRPMNRKPMMYRLWRSPdvpygcegPCKVQSFE-------ARHDVK---- 80  Cowpea golden mosa...
ABU24276   20 RRRLNFET------AIvpyTGNAVPIAARSYVPVSRGVRMKRK-RGDripkgcvgPCKVQDYE-------FKMDVP---- 81  Sweet potato leaf ...
P03565      8 SRNLQRKW-NSNITNRypiKRKYVAGHTRPC---VRRRLLYEPVERP-----------FGHNVlcekqhgDVFNLQq--- 69  African cassava mo...
Q06927      7 RRGLSYVP-RRYNPRNygfKRTFVVKRGDAK---RRQTQVKKLTEDV----------KMSSQRihenqygPEFVMAh--- 69  Pepper huasteco ye...
Q96706      7 RRGVSYSQ-RRFVSRNqssKRGTFVRRTDGK---RRKGPSSKAHDEP----------KMKLQRihenqygPEFVMTh--- 69  Cabbage leaf curl ...
P0CK41      7 KRGSSNYQ-RRGYSRYqgfRRTAIVTRHDGK---RRQHQSNKSNEDP----------KMLVQCirenqfgPDFVMSh--- 69  Bean golden yellow...
P21935      7 RRGRSQTQ-RGSHVRRtgvKRSYGAARGDDR---RRPNVVSKTQVEP----------RMTIQRvqenqfgPEFVLSq--- 69  Squash leaf curl v...
Q66215      5 RRGRPNVQ-RVSHLRRtyfKRPYYQTRGDEK---RRPTAVNKTHDDT----------KMSLQRlhedqfgPDFVLGh--- 67  Squash leaf curl v...
O36455      7 KRGATFVA-RRSYSRNnlfKRSTISKRDDGR---RRSVNATKPNDEP----------KMTAQRmhenqfgPDFVMAh--- 69  Chino del tomate v...
O56373      9 KRGYYFPN-RRFTTRNtvfNRSTAGKRHDGR---RRGGRSVKTSDEP----------KMSAQSihenqygPDFVMSh--- 71  Sida golden mosaic...
Q06661      7 KRGFSYSN-RRFNSRNivfNRPVSGKRHDGK---RRGGNFVKPNDEP----------KMLAQRihenqygPEFVLAh--- 69  Tomato mottle viru...
Q67583      7 KRGATTTS-RRSYSRYplsRRSYSVNRINGK---RGSNIHRKAHDDG----------KMLAQRlhenqfgPEFVMAh--- 69  Bean golden mosaic...
P03564      7 RRGFLANQ-GRGYPRHstgKRSRNVSRIDFK---RRSSKYVHGNDDS----------KMANQRihenqfgPEFVMVh--- 69  Tomato golden mosa...
P27268      7 RRASFCSQ-PRTYPRNsliRQQSLFKRNVSK---RRPFQTVKMVDDS----------MMKAQRihenqygPDFSLAh--- 69  Potato yellow mosa...
Q88947     35 RRVGNRVY-GMPFGSTtsvRRPIRST--------VRRNLFSDQSSSG----------NKSRKTieevhdgSDYLLGn--- 92  Tomato yellow leaf...
O41331     24 RRSVYPAN------RIyngNRSFRLWKSRKSQNWKRYGSTNSVARSPtelfgdpiSKQYTRKEicetqegSEYVLHn--- 94  Tomato leaf curl N...
Q88886      5 RKLTSYFPq---------sKRFRGAKSGMAVVKTSASRRLYKKGKRK--------PDRIKTYT---------YAFSsict 58  Tomato pseudo-curl...
P29073      3 QKRKd-------------lRRSDAGSAVRAKLHKASTGKAFPVGKRA--------PLQIRSYAwetpataTTPSGPis-i 60  Miscanthus streak ...
CAL30139    5 KSKGKRKRd---------eGESSGRWKGAVYKRRKTQYKVVPVRPP---------ALCVLRQQwit-pdkQSVVVT---- 61  Oat dwarf virus
Q89238      8 RGKGKRKLe---------eGESSGRWKGAVYKRRKQAYKVVPVKPP---------ALCVFRYNwln-sdrTNIVVG---- 64  Wheat dwarf virus
O39520      8 KKRKRSGG----------------DSKAKSRGSSSYIPRSVSSRRE---------SLQVATFSwts--sgSGIKFS---- 56  Bean yellow dwarf ...
P31616     12 RKRGRYGKt---------yQALGVKSQRELEQLVNQPGRPVSNRRP---------ALQVAEYLwtt--nkTGMTFS---- 67  Tobacco yellow dwa...
P14985      9 RKRPSSSS-----------------AQASKKRRVYRPAVSRSLARRE--------PLQVQDFVw-----dTDVAFN---- 54  Chloris striate mo...
Q76QL8      6 RKRGDDSNw----------NKRVSKKKPSSA-GLKRAGS--KADRP---------SLQIQTLQha---gtTMITVP---- 56  Maize streak virus...
Q67566      7 RKRTDEGSg----------SRRFTKKKSA---AKGRTSSS-RAIRP---------ALQIQSFVsa---gaATIAVP---- 56  Digitaria streak v...
Q89551      8 RKRSDETGr----------RKRSSGVKQGRT-SAARAGSAVRRTRP---------SLQIQTLQaa---gtSMIEVP---- 60  Sugarcane streak v...
O56311      8 RKRADEVAw----------SKRGTKKKPERT-SAARAGPS-RRIRP---------PLQIATFVaa---gqSMVSVP---- 59  Sugarcane streak E...
Q84369      7 RKRSDEVAw----------SRRKPVKKQDTGFPLPRAGPSVRRGLP---------ALQIQTLTaa---gdTMITVP---- 60  Panicum streak vir...
Q916F7     18 RRRLNFDSpYSSRAAVp-tVRVT-KRQAWTNRPMNRKPRWYRMFKSPdvprgcegPCKVQSFE-------SRHDVV---- 84  Indian cassava mos...
Q9QD50     18 RRRLNYDSpGTSRAAVl-tALGINRKKSWTSRPMYRKPRIYRMYRTAdvprgcegPCKIQSFE-------SRHDIA---- 85  Tomato yellow leaf...
Q9PY63     18 RRRLNFDSpYVSRAAAp-tVLVTNKRRSWANRTMYRKPRIYRMYKSPdvprgcegPCKVQSYE-------QRHDVA---- 85  Pepper leaf curl v...
ABS29524   18 RRRLNFDSpYTSRATVp-tALGTSKRRSWTYRPMYRKPRMYRMYRSPdvprgcegPCKIQSFD-------QRDSVT---- 85  Okra yellow crinkl...
Q8V0I4     18 RRRLNFDSpMQSGVRAp-tVLVTNKRRAWSNRPNYRKPRMYRMYRSPdvpkgcegPCKVQSYE-------QRDDVK---- 85  Hollyhock leaf cru...
Q64IB6     18 RRGLNFDSpYRNRATAp-tVHVTNRKRAWVNRPMYRKPMMYRMYRSPdiprgcegPCKVQSFE-------QKDDVK---- 85  African cassava mo...

consensus  65 NTGKVSLISDPTRGKG-ETNRVRKYIKLKSLYFKGTVWIDENIKDKNMT-------GVFSFWLVRDRRPHGnTPG----T 132
Q08595     73 NNYKSSYISMPVKTRAlSDNRVGDYIKLVNISFTGTVCIKNSQMESDGSpml-glhGLFTCVLVRDKTPRIySATepliP 151 Indian cassava mos...
Q17ZR1     85 HMGKVICLSDVTRGTG-LTHRVGKRFCVKSIYFVGKIWIDENIKVKNHT-------NTVMFWVVRDRRPTG-TPL----D 151 Sida yellow vein v...
Q6ZX39     86 HMGRVICLSDVTRGVG-LTHRVGKRFCVKSLYFVGKIWMDENIKVKNHT-------NTVMFWIVRDRRPSG-TPS----D 152 Tomato leaf curl C...
Q1JRP0     85 HTGKVICVSDVTRGNG-ITHRVGKRFCIKSIYVIGKIWMDENIKVKNHT-------NTVMFFLVRDRRPSG-TPM----D 151 Tomato yellow leaf...
Q8JNG6     86 HTGEVLCVSDVTRGNG-LTHRVGKRFCIKSIYIVGKVWMDENIKVKNHT-------NTCMFWLVRDRRPVT-TPY----G 152 Tomato leaf curl S...
Q6L6V9     86 HSGKMLCISDITRGNG-LTHRVGKRFCIKSVYIIGKIWMDENIKLKNHT-------NNVIFWLVRDRRPVS-VPY----G 152 Honeysuckle yellow...
AWW16572   80 HVGKVMCISDVTRGNG-ITHRVGKRFCVKSVYILGKIWMDENIKLQNHT-------NSVMFWLVRDRRPYG-TPM----D 146 Squash leaf curl v...
O55376     81 HTGTFQCCSDVTRGMG-LTHRVGKRFTIKTIGIWGKIWMDDNIKLKNHT-------NIVIFFLVRDRRPSG-EPV----S 147 Cowpea golden mosa...
ABU24276   82 HTGTFACVSDFTRGTG-LTHRLGKRVCIKSMGIDGKVWMDDNVAKRDHT-------NIITYWLIRDRRPNK-DPL----T 148 Sweet potato leaf ...
```

```
P03565      70 NTSYTSFVTYPSRGPS-GDGRSRDYIKLQSMSVSGVIHAKANGNDDPMEvsp-vvnGVFVFSLIMDTKPYLpAGVqglpT 147 African cassava mo...
Q06927      70 NTAISTFINYPQLCKT-QPNRSRSYIKLKSLHFKGTLKIERVGSEVNMAglnpkieGVFTVVLVVDRKPHLnPTGnl-lQ 147 Pepper huasteco ye...
Q96706      70 NSALSTFINFPVLGKI-EPNRSRSYIKLNRLSFKGTVKIERVHADVNMDgviskieGVFSLVIVVDRKPHLsSTGgl-hT 147 Cabbage leaf curl ...
P0CK41      70 NTAISTFINYPQLGKI-EPNRCRSYIKLKRLRFKGTVKIERMHTDVNMDglspkieGVFSIVIVVDRKPHLsPSGcl-hT 147 Bean golden yellow...
P21935      70 NSALSTFVTYPSYVKT-VPNRTRTYIKLKRVRFKGTLKIERGQGDTIMDgpssnieGVFSMVIVVDRKPHVsQSGrl-hT 147 Squash leaf curl v...
Q66215      68 NTALSTFITYPSIVKT-EPNRTRSYIKLKRLRFKGTLKIERGHGGTVMEdptskieGVFSMVVVVDRKPHVnPSGrl-hT 145 Squash leaf curl v...
O36455      70 NSALETFISFPCLGKT-QPNRTRSYIKLKRLRFKGTVKIERVTSDMNMDgstpkveGVFSLVVVVDRKPHLgGSGsl-hT 147 Chino del tomate v...
O56373      72 NSAISTFISYPDLGKI-EPGRSRSYIKLKRLRFKGTVKIERVTSDVNMDgsapkveGVFSLVVVVDRKPHLtASGgl-hT 149 Sida golden mosaic...
Q06661      70 NSAISTFIPVIHLGQVpKPSRSRSYIKLKRLRFKGTVKIERVQSDLNMDgfmpkveGVFSMVVVVDRKPHLgPSGcl-hT 148 Tomato mottle viru...
Q67583      70 NSAISTFINFPSLGKT-EPNRSKSFIKLKRLRFKGTVKIERV---LNMDgstpkieGVFSMVLVVDRKPHLgSSGcl-hT 144 Bean golden mosaic...
P03564      70 NTAISTFITFPSLGKT-EPSRSRSYIKLKRLRFKGTVKIERVHVDLSMDgpspkieGVFSLVVVVDRQPHLsPTGcl-hT 147 Tomato golden mosa...
P27268      70 NTAVSTFISYPDIAKS-LPNRTRSYIKLKRLRFKGIVKVERVHVEVNMDcsvpkteGVFSLVIVVDRKPHLgPSGgl-pT 147 Potato yellow mosa...
Q88947      93 NTSKVSYISYPPLSRSeFGNRLDAFVKILGFNVSGSVAVR--HLEQRATgasqgihGIYCTAVVRDKRPCQfSAVepivP 170 Tomato yellow leaf...
O41331      95 NRYMTSYVTYPAKTRTgTNNRVRSYIKLKSLNISGTFAVRTSDMMTEVGqtn-glyGVMSLVVVRDNSRLSaTTY----T 169 Tomato leaf curl N...
Q88886      59 DKGTIVYMNSWSLGLG-PQQRSSDIEILKSMYIRLTVALSENAASQVKT-------YVVKWALIVDQVPGEtLVG----V 126 Tomato pseudo-curl...
P29073      61 TDGTIFMCNTIDPGTG-DDQRSRHTTMLYKMSLNVVLWPGATTAQIVGP-------FRVNFWLVYDAAPTGvVPK----L 128 Miscanthus streak ...
CAL30139    62 NTPRIDLITCFAAGKG-DDNRHTNQTIMYKFHLQGSVYVADASSKFVTP-------LRLYHWLVYDAEPKQaMPG----I 129 Oat dwarf virus
Q89238      65 NTPRVDLITCFAQGKA-DNNRHTNQTVLYKFNIQGTCYMSDASAPFIGP-------VRLYHWLVYDAEPKQaMPD----A 132 Wheat dwarf virus
O39520      57 PGGAAYLVSNFPQGAN-DNCRHTNKTVLYKFMAKNTIYLDSSHYMKVFR-------APFHFWLVYDKSPGAnVPS----T 124 Bean yellow dwarf ...
P31616      68 PGGSTFLITNFPQGAN-ENCRHTNRTITYKMAVKTWVALDGSMFSRVSK-------FPIYFWLVYDKNPGEsNPS----P 135 Tobacco yellow dwa...
P14985      55 RGGGCYLLTSYARGSA-ENQRKTAETITYKVAVNLGCAISGTMQQYCIS-------SRPVCWIVYDAAPTGsAVT----P 122 Chloris striate mo...
Q76QL8      57 SGGVCDLINTYARGSD-EGNRHTSETLTYKIAIDYHFVADAAACRYSNT-------GTGVMWLVYDTTPGGqAPT----P 124 Maize streak virus...
Q67566      57 TGGVCHLLSSYSRGSG-EGDRHTNETVTYKAAFDYHFSANAGPCAYSSI-------GVGVLWLVYDAQPSGqVPA----V 124 Digitaria streak v...
Q89551      61 SGGVCDLLGSFSRGSD-EGNRHTNETVIYKVALDYHFIATAAACKYSSI-------GTGVVWLVYDAQPSGnPPT----V 128 Sugarcane streak v...
O56311      60 SGGVCELLGSYARGAD-EANRHTNETVTYKVALDYHFVATAAACKYSSI-------GTGVAWLVYDAQPTGnTPT----T 127 Sugarcane streak E...
Q84369      61 SGGICSLIGTYARGSD-EGNRHTNETLTYKVALDYHFVATAAACKYSSI-------GVGVMWLVYDAQPTGnSPE----V 128 Panicum streak vir...
Q916F7      85 HIGKVMCISDVTRGVG-LTHRVGKRFCVKSVYILGKIWMDENIKTKNHT-------NSVMFFLVRDRRPVD-KPQ----D 151 Indian cassava mos...
Q9QD50      86 HTGKVMCVTDVTRGGG-LTHRTGKRFCVKSLYILGKIWMDENIKTKNHT-------NTVMFYVVRDRRPYG-TPQ----D 152 Tomato yellow leaf...
Q9PY63      86 HVGKVICVSDVTRGNG-LTHRVGKGPVIKSVYVWGRIWMDE-ISSQESH-------QSVMFFLVRDRRPFG-TPQ----D 151 Pepper leaf curl v...
ABS29524    86 HTGNSRCLSDVTRGNG-LTHRTGKRFVIKSVYILGKVWMDENVKKSNHT-------NTCMFWLVRDRRPYGnSPQ----D 153 Okra yellow crinkl...
Q8V0I4      86 HTGIVRCVSDVTKGTG-LTHRTGKRFTVKSIYILGKIWMDENIKKQNHT-------NNVMFFLVRDRRPYGnSPM----D 153 Hollyhock leaf cru...
Q64IB6      86 HLGICKVISDVTPGPG-LTHRVGKKFCIKSIYILGKIWMDENIQKRNPP-------NNGIFYLLRDRRPYGnAPQ----D 153 African cassava mo...

consensus 133 FDELFGM---YDNEPGTLTVKRDlrdRFVVKRKWKRVVSSEGdtnm--------vdfPGKNQLSvRRFFKWLGVKDEW-N 200
Q08595    152 FPQLFGS---INASYADLSIQDPykdRFTVIRQVSYPVNTEKgdhm--------crfKGTRRFG-GRYPIWTSFKDDGgS 219 Indian cassava mos...
Q17ZR1    152 FQQVFNV---YDNEPAMLLSRTT---SVIVFRFYEGFMQRSP---------------EGNMQLRsRRLSESSIVLTIM-L 209 Sida yellow vein v...
Q6ZX39    153 FQQVFNV---YDNEPSTATVKNDqrdRYQVIRRFQATVTGGQy--------------AAKAQAIiRRFYR-VNNYVVY-N 213 Tomato leaf curl C...
Q1JRP0    152 FQQVFNC---YDNEPSTATVKNDlrdRFQVRRKFYSTVTGGQy--------------ACKEQALvKRFIR-VNTHVVY-N 212 Tomato yellow leaf...
Q8JNG6    153 FGELFNM---YDNEPSTATIKNDlrdRCQVLKRFTASLSGGQy--------------ASKEQCViRRFYK-IYNHIVY-N 213 Tomato leaf curl S...
Q6L6V9    153 FQEAFNM---YENEPSTATIKQDlrdRLQVLHKFTGTVTGGQy--------------ASKEQAMiKRFWR-LNHHVTY-N 213 Honeysuckle yellow...
AWW16572  147 FGHVFNM---FDNEPSTATVKNDlrdRYQVLHKFYGKVTGGQy--------------ASNEQAIvKRFWK-VNNHVVY-N 207 Squash leaf curl v...
O55376    148 FGSLFNM---FDNEPTTATVKQEyrdRFQVMRRFHASVTGGQy--------------ASKEQALvKRFFRNINHRVVY-N 209 Cowpea golden mosa...
ABU24276  149 FVQAFTM---YDNEPTTAKIRMDlrdRMQVLKKFSVTVSGGPy--------------NHKEQALvRKFFKGLYNHVTY-N 210 Sweet potato leaf ...
P03565    148 FEELFGP---YSACYVNLRLLNNqqhRYRVLHSVKRFVSSSGdtkv--------sqfRFNKRLStRRYTIWASFHDGD-L 215 African cassava mo...
Q06927    148 FDELFGA---RIHSLGNLAVTPAlkeRFYILHVLKRVISVEKdsmm--------ldlEGSTCLSsRRYNCWSTFKDLD-P 215 Pepper huasteco ye...
Q96706    148 FDEIFGA---RIHSHGNLAITPGlkdRYYVLHVLKRVLSVEKdtlm--------vdlEGSTTISnRRYNCWASFNDLE-H 215 Cabbage leaf curl ...
P0CK41    148 FDELFGA---RINSHGNLAVMPSlkdRFYIRHLLKRVLSVDKdttm--------idvEGSTLLSnKRYNMWSTFNDFD-H 215 Bean golden yellow...
P21935    148 FDELFGA---RIHCHGNLSVVPAlkdRYYIRHVTKRVVSLEKdtll--------idlHGTTQLSnKRYNCWASFSDLE-R 215 Squash leaf curl v...
Q66215    146 FDELFGA---RIHSHGNLAIVPAlkdRFYIKHVMKRVLSVEKdtlm--------vdlQGSTTFSnKRFNCWSAFNDLE-R 213 Squash leaf curl v...
O36455    148 FDELFGA---RIHSHGNLSIPPSlkdRFYIRHVFKRVLSVEKdsmm--------vdvEGSTALSnRRFNCWSTFKDLD-R 215 Chino del tomate v...
O56373    150 FDELFGA---RIHSHGNLSITPSlkdRFYVRHVFKRVLSVEKdtlm--------vdvEGSTALSnRRINCWSTFKDLE-R 217 Sida golden mosaic...
Q06661    149 FDELFGA---RINSHGNLTIVPSlkdRFYIRHVFKRVLSVEKdtlm--------vdvEGSTTLSnRRYNCWSTFKDLD-R 216 Tomato mottle viru...
Q67583    145 FDELFGA---RIHSHGNLAITATlkeRFYIRHVYKRVLSVEKdsmm--------vdvEGSTYLSsRRFNCWATFKDDE-R 212 Bean golden mosaic...
P03564    148 FDELFGA---RIHSHGNLAVSSAlkdRFYIRHVFKRVISVEKdstm--------idlEGMTSFTnRRFNCWSAFKDFD-R 215 Tomato golden mosa...
```

```
P27268     148 FDELFGA---RIHSHGNLAIVPSlkdRFYIRHVLKRVISVEKdtmm--------vdiEGVVALSsRRFNCWAGFKDLD-I 215 Potato yellow mosa...
Q88947     171 FPELFGL---EKMACSSLRVRDIhrsRFSLVYQKKVVVNSSLpth----------vfKFNYSVKfNRFPFWVSFKDTS-D 236 Tomato yellow leaf...
O41331     170 FVELFCS---VNACRGRLKVAERhhdRFVLLNPTSIVVNTPHpnti--------kkfCIRNCIP-RTYTTWVTFKDEEeD 237 Tomato leaf curl N...
Q88886     127 AD-VYKTcpsPYPYVQCAYIADDnhsRFQVLRSGFLSLSGNGlavgss---trtgcpAMKNMASiNKFCKNLNVRCVYdA 202 Tomato pseudo-curl...
P29073     129 TD-IFDVa--YPKWGNTWQVSRSnvhRFIVKRKWKVDYQSSGvpvgkrq-ssgveyaPVNNVVEcNKFFEKLRVKTEW-A 203 Miscanthus streak ...
CAL30139   130 ED-IFTMp--WNVLPSTWTIWRAwahRFVVKRKWHVDLVSDGkkvgsstadtrynyvVGKNIVDcNKFIKGLRVSTEW-M 205 Oat dwarf virus
Q89238     133 TD-IFTMp--WNLLPSTWTVQRAwshRFVVKRKWTVNLVTDGrkvgsktvdqrynwvVGKNIVDaSKFFKGLRVTTEW-M 208 Wheat dwarf virus
O39520     125 GD-IFEGpslFPHNPWTWTVSRAachRFVVKKTWSCVVESNGidptkgqgatyygpgPCNQVKScNKFFKRLGVSTEW-K 202 Bean yellow dwarf ...
P31616     136 SA-IFDSl--YQDQPGTWTVTRNvchRFVVKKTWSCMLESNGvdpnakqgssyygpgPCYQWRHvTKFFKRLGVSTEW-K 211 Tobacco yellow dwa...
P14985     123 KD-IFGYpegLVNWPTTWKVARAvshRFIVKRRWVFTMESNGsrfdrdy--tnlpaaIPQSLPVlNKFAKQLGVRTEW-K 198 Chloris striate mo...
Q76QL8     125 QT-IFAYpdtLKAWPATWKVSRElchRFVVKRRWLFNMETDGrigsdip-psnaswkPCKRNIYfHKFTSGLGVRTQW-K 201 Maize streak virus...
Q67566     125 TD-IFPHdttLQSFPYTWKVGREvchRFVVKRRWCFTMETNGrigsdkp-psnavwpPCKRSIYfHKFATGLGVKTEW-K 201 Digitaria streak v...
Q89551     129 KD-IFPHpdtLTAFPYTWKVGREvchRFVVKRRWTFTMEVNGrigsdip-pstscwpPCRKNIYfHKFVTGLGVKTEW-K 205 Sugarcane streak v...
O56311     128 KD-IFGYsdsLVAFPYTWKVSREvchRFVVKRRCTFTMETDGrigsdvp-pantswsPCKRAIYfHKFFTGLGVKTEW-K 204 Sugarcane streak E...
Q84369     129 KD-IFPHsdtLSAFPYTWKVGREvchRFVVKRRWCFTMETNGrigsdvp-pantawpPCKKDIYfHKFCTGLGVKTEW-K 205 Panicum streak vir...
Q916F7     152 FGEVFNM---FDNEPSTATVKNMhrdRYQVLRKWSATVTGGQy--------------ASKEQALvRRFFR-VNNYVVY-N 212 Indian cassava mos...
Q9QD50     153 FGQVFNM---FDNEPSTATVKNDlrdRFQVLRKFTATVVGGQy--------------ACKEQTLvRKFMR-LNNYVVY-N 213 Tomato yellow leaf...
Q9PY63     152 FGQVFNM---YDNEPSTATVKNDnrdRFQVLRRFQATVTGGQy--------------ASKEQAIvRKFMK-VNNHVTY-N 212 Pepper leaf curl v...
ABS29524   154 FGQVFNM---FDNEPSTATVMNDkrdRYQVLKRFQATVTGGPs--------------GCKEAAIvRRFFK-INHHVTY-N 214 Okra yellow crinkl...
Q8V0I4     154 FGQVFNM---FDNEPSTATIKNDyrdRFQVMRKFSATVTGGPs--------------GMKEQALvRRFFK-INSHTVY-N 214 Hollyhock leaf cru...
Q64IB6     154 FGQIFNM---FDNEPSTATIKNDlrdRFQVLRKFHATVVGGPs--------------GMKEQALvKRFYR-LNHHVTY-N 214 African cassava mo...

consensus 201 NSETGKYGNIKKNALLLYYCWMSAPNSGASTTki--------rVYFKLSYNG 244
Q08595     220 GDSSGLYSNTYKNAILVYYVWLSDVSSQLEMY-----------CKYVTRYIG 260 Indian cassava mosaic virus
Q17ZR1     210 FTTTRRRQSMRTTPRMLCYCTWHVLMPLTQCMrllnvevistiLSAIKINKV 261 Sida yellow vein virus
Q6ZX39     214 HQEAGKYENHTENALLLYMACTHASNPVYATLkv--------rSYFYDSVTN 257 Tomato leaf curl China virus
Q1JRP0     213 HQEQAKYENHTENALLLYMVSTHASNPVYATLkv--------rIYFYDSQMN 256 Tomato yellow leaf curl China virus
Q8JNG6     214 HQEQGKYENHTENALLLYMACTHASNPVYATLki--------rVYFYDSISN 257 Tomato leaf curl Sudan virus - [Gezira]
Q6L6V9     214 HQEAAKYANHTENALLLYMACTHASNPVYATLki--------rVYFYDSVQN 257 Honeysuckle yellow vein mosaic virus
AWW16572   208 HQEAGKYENHTENALLLYMACTHASNPVYATLki--------rIYFYDSLMN 251 Squash leaf curl virus
O55376     210 QQEGAEYKNHHENALMLYMACSHASNPVYATIkv--------rIYFYDSITN 253 Cowpea golden mosaic virus-[Nigeria]
ABU24276   211 HKEEAKYENHLENALMLYSASSHASNPVYQTLrc--------rAYFYDSHNN 254 Sweet potato leaf curl virus
P03565     216 VNAGGNYRNISKNAILVSYAFVSEHAMSCKPF-----------VQIETSYVG 256 African cassava mosaic virus - [West Kenya 844]
Q06927     216 SSCNGVYDNISKNAILVYYCWMSDAMSKASTF-----------VSFDLDYFG 256 Pepper huasteco yellow vein virus
Q96706     216 DLCNGVYANISKNAILVYYCWMSDAMSKASTF-----------VSYDLDYLG 256 Cabbage leaf curl Jamaica virus
P0CK41     216 DSCNGVYANIAKNALLVYYCWMSDIMSKASTF-----------VSYDLDYVG 256 Bean golden yellow mosaic virus-[Puerto Rico]
P21935     216 DSCNGVYGNITKNALLVYYCWLSDAQSKASTY-----------VSFELDYLG 256 Squash leaf curl virus
Q66215     214 DSCNGVYANISKNAILVYYCWMSDTPSKASTY-----------VSFDLEYVG 254 Squash leaf curl virus
O36455     216 DSCNGVYGNISKNALLVYYCWMSDTMSRVSSF-----------VSFDLDYIG 256 Chino del tomate virus
O56373     218 DSCKGVYGNVSKNALLVYYCWMSDTVSKASSF-----------VSFDLDYVG 258 Sida golden mosaic virus
Q06661     217 ESCKGVYDNISKNALLVYYCWMSDTPANASSF-----------VSFDLDYIG 257 Tomato mottle virus-[Florida]
Q67583     213 DSCNGVYANISKNALLVYYCWMSDAMSKASTF-----------VSFDLDYVG 253 Bean golden mosaic virus-[Brazil]
P03564     216 QACNGVYGNISKNAILVYYCWMSDIVSKASTF-----------VSFDLDYVG 256 Tomato golden mosaic virus-Yellow vein
P27268     216 ESRKGVYDNINKNALLVYYCWMSDTVSKASTF-----------VSFDLDYIG 256 Potato yellow mosaic virus - [Venezuela]
Q88947     237 SEPSGRYSNVSKNALIVYYVWLCDTNVTADSY-----------VKYDLNYIG 277 Tomato yellow leaf curl Thailand virus-[1]
O41331     238 SCCTGRYSNTLRNAIILYYVWLSDVSSQVDLY-----------SNVILNYIG 278 Tomato leaf curl New Delhi virus-[Lucknow]
Q88886     203 DSATGDIASIKRGAVYLVIWPDVEIRYGFSCT------------MYHRNGNA 242 Tomato pseudo-curly top virus
P29073     204 NTSTGAIGDVKKGALYLCANTRQMPAGDSVTTscttmmqgstrLYFKVLGNQ 255 Miscanthus streak virus - [91]
CAL30139   206 NTGDGKIGDIKKGALYLVTCTRAGVTGDSASTgfsvvcnlthaCYFKSIGLQ 257 Oat dwarf virus
Q89238     209 NTGDGKIGDIKKGALYLISSTRGGVTGDSASTafdvvcaythaCYFKAIGIQ 260 Wheat dwarf virus
O39520     203 NSATGDVGDIKEGALYIVG----APSQKSDVYvng-----yfrVYFKSVGNQ 245 Bean yellow dwarf virus
P31616     212 NSSTGDVADIKEGALYLVC----APGGGATVRvgg-----rfrMYFKSVGNQ 254 Tobacco yellow dwarf virus (strain Australia)
P14985     199 NAEGGDFGDIKSGALYLVM----APANGAVFVarg-----nvrVYFKSVGNQ 241 Chloris striate mosaic virus
Q76QL8     202 NVTDGGVGAIQRGALYMVI----APGNGLTFTahg-----qtrLYFKSVGNQ 244 Maize streak virus - [Reunion2]
```

```
Q67566     202 NTTDGGVGSIKKGALYFVI----APGSGIDFTlfg-----tcrMYFKSVGNQ 244 Digitaria streak virus
Q89551     206 NTTGGEVGDIKKGALYIVI----APGNGLDFTvhg-----narLYFKSVGNQ 248 Sugarcane streak virus - [Natal]
O56311     205 NLTDGGVGAIKKGALYLVI----APGNGLEFTchg-----qarLYFKSVGNQ 247 Sugarcane streak Egypt virus - [Giza]
Q84369     206 NVTDGKVGAIKKGALYIVI----APGNGLEFTvhg-----qcrLYFKSVGNQ 248 Panicum streak virus - Karino
Q916F7     213 QQEAGKYENHTENALMLYMACTHASNPVYATLki--------rIYFYDSVSN 256 Indian cassava mosaic virus
Q9QD50     214 HQETAKYENHTENALLLYMASTHASNPVYATLki--------rIYFYDSITN 257 Tomato yellow leaf curl Indonesia virus-[Lembang]
Q9PY63     213 HQEAAKYDNHTENALLLYMACTHASNPVYATLki--------rIYFYDSVQN 256 Pepper leaf curl virus
ABS29524   215 HQEAAKYENHTENALLLYMACTHASNPVYASLki--------rMYFYDSVSN 258 Okra yellow crinkle virus-[Mali]
Q8V0I4     215 HQEAAKYENHTENALLLYMACTHASNPVYATLki--------rIYFYDSVSN 258 Hollyhock leaf crumple virus-[Cairo]
Q64IB6     215 HQEAGKYENHTENALLLYMACTHASNPVYATLki--------rIYFYDSIGN 258 African cassava mosaic virus-Uganda Severe
```

**Supplementary Figure S5.** Alignment of Coat Proteins for Begomoviral and Single amino acid polymorphism (SAPs).
